# Astragalus polysaccharides combined with radiochemotherapy for cervical cancer: a systematic review and meta-analysis of randomized controlled studies

**DOI:** 10.3389/fphar.2025.1699902

**Published:** 2025-11-11

**Authors:** Tao Sun, Ruping Zhao, Yaling Zhang, Dongdong Li, Xishen Shan, Xiaoyu Tang, Yuling Zheng

**Affiliations:** 1 Department of Oncology, The First Affiliated Hospital of Henan University of Chinese Medicine, Zhengzhou, China; 2 Collaborative Innovation Center of Prevention and Treatment of Major Diseases by Chinese and Western Medicine, Henan Province, Zhengzhou, China; 3 The First Affiliated Hospital of Henan University of Chinese Medicine, Zhengzhou, China; 4 Department of Oncology, Henan Province Hospital of TCM, Zhengzhou, China

**Keywords:** Astragalus Polysaccharide Injection (APS), cervical cancer, meta-analysis, systematic review, randomized controlled trials (RCTs), trial sequential analysis (TSA)

## Abstract

**Backgrounds:**

Cervical cancer ranks among the most common malignant tumors affecting women globally. Currently, radiotherapy and chemotherapy are commonly used treatments for cervical cancer, yet they are often accompanied by severe toxic side effects. Therefore, enhancing the efficacy of radiotherapy and chemotherapy while mitigating their adverse reactions has become a critical challenge in contemporary cervical cancer treatment. Exploring or developing novel combination therapy regimens has also emerged as a significant research direction. Astragalus Polysaccharides, a natural extract derived from the traditional Chinese medicinal herb Astragalus membranaceus (Huang Qi), is currently widely used in China as an adjunctive therapy for various cancers.

**Objective:**

This systematic review aims to determine the clinical efficacy and safety of Astragalus Polysaccharide Injection (APS) combined with chemoradiotherapy for cervical cancer based on existing data.

**Methods:**

Systematic searches were conducted in PubMed, Web of Science, Embase, Cochrane Library, China National Knowledge Infrastructure (CNKI), Wanfang Data, Chinese Scientific Journal Database (VIP), SinoMed, and China Clinical Trials Registry Platform. The search period spanned from the inception of each database to August 1, 2025. The ROB2 tool was used to evaluate the quality of included RCTs. Meta-analysis was performed using Review Manager software, and sensitivity analysis was conducted with Stata 18 software. Evidence quality was assessed using the GRADE system. Additionally, TSA was employed to calculate the final required sample size for this meta-analysis and to validate the reliability of conclusions. The protocol for this systematic review was registered and published in PROSPERO (CRD420251139830).

**Results:**

This study included 9 RCTs involving 776 patients. Meta-analysis results showed that compared with chemoradiotherapy alone, APS combined with chemoradiotherapy improved the objective response rate (ORR, RR = 1.43, 95% CI: 1.24–1.64) and disease control rate (DCR, RR = 1.16, 95% CI: 1.08–1.24), and Karnofsky Performance Status (KPS) score (MD = 6.64, 95% CI: 4.12–9.16). It also modulates immune function: CD3^+^ T lymphocyte ratio (MD = 14.51, 95% CI: 1.64–27.39), CD4^+^ T lymphocyte ratio (MD = 4.87, 95% CI: 1.79–7.96), and CD4^+^/CD8^+^ ratio (MD = 0.25, 95% CI: 0.17–0.33). Furthermore, APS combined with chemoradiotherapy further reduced tumor marker levels, including: CEA (MD = −1.24, 95% CI: −1.58 to −0.89), SCC (MD = −1.18, 95% CI: -1.51 to −0.84), and CA125 (MD = −9.12, 95% CI: −18.22 to −0.01). Subgroup analyses of ORR and DCR suggest that APS can enhance the clinical efficacy of radiotherapy and chemotherapy for cervical cancer, respectively. TSA indicated that the results for ORR, DCR, and ADRs were certain, with other trials unlikely to alter the findings.

**Conclusion:**

APS combined with chemoradiotherapy improves response rates, enhances immune function, and reduces treatment-related toxicity in cervical cancer; however, confirmation through larger, high-quality multicenter RCTs is warranted.

**Systematic Review Registration:**

https://www.crd.york.ac.uk/PROSPERO/recorddashboard, identifier CRD420251139830.

## 1 Introduction

According to GLOBOCAN 2022, cervical cancer is the fourth most common female malignancy worldwide, ranking fourth in both incidence and mortality after breast, lung, and colorectal cancers (Bray et al., 2024). Persistent infection with high-risk human papillomavirus (HPV) is the primary cause of cervical cancer, with HPV types 16 and 18 accounting for approximately 70% of cases (Molina et al., 2024; Arbyn et al., 2011). Despite improved early screening, global incidence and mortality of cervical cancer remain high, imposing a growing disease burden, especially in less developed regions (Wentzensen and Arbyn, 2017; Li et al., 2025a; Small et al., 2017; Bedell et al., 2020).

Currently, radiotherapy and chemotherapy serve as the core treatment modalities for advanced or recurrent cervical cancer. Concurrent surgery with radiotherapy and chemotherapy can also enhance the success rate of surgical resection and reduce the risk of postoperative recurrence (Abu-Rustum et al., 2023; Schubert et al., 2023; Burmeister et al., 2022). Although radiotherapy and chemotherapy effectively reduce tumor burden and prolong survival, treatment-related adverse events—such as impaired immune function, gastrointestinal reactions, bone marrow suppression, and hepatic/renal toxicity—remain major concerns (Puspitasari et al., 2021). These toxicities frequently compromise patient tolerance and treatment completion rates, leading to treatment delays or interruptions that may ultimately impact prognosis (Palagudi et al., 2024; Federico et al., 2021; Pourhanifeh et al., 2020). Existing supportive therapies offer only limited relief (Christiansen et al., 2020). Consequently, there is an urgent need to develop effective adjunctive therapies that enhance the efficacy of chemoradiotherapy, mitigate related adverse reactions, and simultaneously improve treatment adherence and quality of life.

Astragalus Polysaccharide Injection (APS), a standardized Chinese herbal medicine (Approval Number from the China National Medical Products Administration: Z20040086), is widely used in China as an adjuvant treatment for various malignant tumors (Li et al., 2025b). As the main active component of Astragalus membranaceus (Huang Qi), APS exhibits diverse pharmacological properties, including antitumor, antioxidant, anti-inflammatory, and immunomodulatory activities (Zhang and Feng, 2022; Zhang et al., 2025; Shi and Ma, 2024). Its antitumor mechanisms involve enhancing cellular and humoral immunity, inhibiting tumor growth, increasing sensitivity to radiotherapy and chemotherapy, and reducing treatment-related toxicity (Li et al., 2020a; Zhou et al., 2017; Wang et al., 2024; Xu et al., 2023; He et al., 2024). In a systematic review, Li et al. reported that APS combined with conventional antitumor therapy improved clinical efficacy, enhanced immune function, and demonstrated a favorable safety profile (Li et al., 2025b). Furthermore, several experimental studies have suggested the potential utility of APS in the treatment of cervical cancer (Xia et al., 2020; Zhai et al., 2018; Elham et al., 2021; Liu et al., 2023). However, no comprehensive meta-analysis has yet evaluated the combined use of APS and chemoradiotherapy specifically in cervical cancer. Therefore, this study conducted a meta-analysis to comprehensively evaluate the clinical efficacy and safety of APS as an adjunctive therapy combined with chemoradiotherapy in cervical cancer patients, comparing it with conventional therapy alone. This aims to provide evidence-based guidance and treatment recommendations for clinical practice.

## 2 Methods

### 2.1 Study registration

This systematic review and meta-analysis adhered to the 2020 Preferred Reporting Items for Systematic Reviews and Meta-Analyses (PRISMA) guidelines to ensure methodological transparency and minimize potential biases (Supplementary Material S1) (Page et al., 2021). The study protocol was registered in the PROSPERO database (www.crd.york.ac.uk) under registration number CRD420251139830.

All APS used in the clinical studies included in this research are herbal extract preparations approved for production by the China National Medical Products Administration, with the batch number: Z20040086.

### 2.2 Search strategy

Two researchers (TS and RZ) independently and systematically searched eight Chinese and English electronic databases—PubMed, Web of Science, Embase, Cochrane Library, China National Knowledge Infrastructure (CNKI), Wanfang Data, Chinese Scientific Journal Database (VIP), and SinoMed—to comprehensively collect all RCTs comparing APS combined with radiochemotherapy *versus* radiochemotherapy alone for cervical cancer. Additionally, manual searches were conducted in the China Clinical Trial Registry, relevant review articles, and reference lists of included studies to supplement the identification of eligible studies. The search period spanned from the inception of each database to August 1, 2025, with no language restrictions. The search strategy combined MeSH terms and free-text keywords such as “Astragalus Polysaccharide Injection,” “Astragalus polysaccharide for injection,” “cervical cancer,” “cervical malignant tumor,” and “uterine cervical cancer.” Detailed search strategies and results for each database are provided in Supplementary Material S2.

### 2.3 Eligibility criteria

#### 2.3.1 Inclusion criteria

Based on the PICOS principles, the inclusion criteria for this study are as follows.

1. Participants (P): Patients with pathologically confirmed cervical cancer, regardless of age, gender, or ethnicity.
2. Interventions and Controls (I/C): Patients in the control group received standardized chemotherapy and radiotherapy regimens (referencing CSCO clinical practice guidelines or NCCN guidelines) supplemented with routine symptomatic treatment as needed to manage adverse reactions; The treatment group received APS in combination with the control group’s radiochemotherapy regimen. The specific administration method is as follows: 250 mg of APS administered intravenously once daily during chemotherapy.
3. Outcome (O): Primary efficacy outcomes are short-term survival outcomes, including objective response rate (ORR) and disease control rate (DCR). ORR and DCR are assessed according to World Health Organization (WHO) criteria or Response Evaluation Criteria in Solid Tumors (RECIST) (Eisenhauer et al., 2009), classifying tumor response as complete response (CR), partial response (PR), stable disease (SD), or disease progression (PD). ORR is defined as the sum of CR and PR, while DCR is defined as the sum of CR, PR, and SD. Secondary outcomes include: (1) Immune indicators: CD3^+^ T lymphocyte ratio, CD4^+^ T lymphocyte ratio, CD8^+^ T lymphocyte ratio, and CD4^+^/CD8^+^ ratio; (2) Tumor marker levels: carcinoembryonic antigen (CEA), squamous cell carcinoma antigen (SCC), carbohydrate antigen 125 (CA125); (3) Performance status scores: Karnofsky Performance Status (KPS) score; (4) Blood cell counts: white blood cells, red blood cells, and platelets; (5) And the incidence of adverse drug reactions (ADRs), such as gastrointestinal reactions, myelosuppression, and liver and kidney damage.
4. Study Type (S): The study design is RCTs, without restrictions on blinding status, publication type, or language.

#### 2.3.2 Exclusion criteria

Based on the PICOS principles, the exclusion criteria for this study are as follows.

1. Participants (P): Patients with secondary cervical cancer or those with concurrent other primary malignancies;
2. Interventions and Controls (I/C): Studies where the control or treatment group used additional Chinese herbal formulations alongside APS;
3. Outcomes (O): Studies with incomplete data reporting or where complete data could not be extracted; and duplicated publications;
4. Study types (S): Non-randomized controlled trials, including animal studies, *in vitro* research, reviews, case reports, and letters to the editor.

### 2.4 Study selection and data extraction

The retrieved literature was uniformly managed using EndNote 21.0. After removing duplicates, two researchers (YZ and DL) independently conducted an initial screening of the remaining titles and abstracts based on predefined inclusion and exclusion criteria, excluding studies that clearly did not meet the requirements. Subsequently, full texts of potentially eligible studies underwent detailed assessment to determine final inclusion. Any discrepancies arising during screening were resolved through discussion and consensus between the two researchers. If agreement could not be reached, a third researcher (TS) served as an arbitrator.

Data extraction was performed independently by two researchers (XS and XT) using Microsoft Excel and a pre-designed standardized data extraction form. Extracted information primarily included the following aspects.

1. Basic information: First author, journal of publication, publication year, and study title;
2. Baseline characteristics: Sample size, patient age, clinical stage of cervical cancer, and histological type;
3. Intervention details: Radiotherapy and chemotherapy regimens used in the control group (including specific treatment methods, drug dosages, treatment cycles, and frequency); dosage, treatment cycles, and frequency of APS in the treatment group; detailed descriptions of other adjuvant therapeutic measures in both groups; and overall treatment duration;
4. Outcome measures: All relevant efficacy and safety outcome data reported in the study;
5. Additional information: Diagnostic criteria for cervical cancer, efficacy assessment standards, randomization methods, and specific descriptions of reported adverse reactions.

### 2.5 Risk of bias assessment

Two researchers (TS and RZ) independently assessed the methodological quality of included RCTs using the ROB 2.0 tool (Sterne et al., 2019). In cases of disagreement, the final decision was made by an arbitrator (YZ). Key domains evaluated included: (1) randomization process, (2) deviations from intended interventions, (3) missing outcome data, (4) measurement of the outcome, and (5) selection of the reported result. Risk of bias was assessed as “low,” “some concerns,” or “high” according to the ROB2 criteria. This determination was based on responses to a series of questions within each domain, with possible answers including: “Yes” (Y), “Probably Yes” (PY), “Probably No” (PN), “No” (N), and “No Information” (NI).

### 2.6 Statistical analysis

All statistical analyses were performed using Review Manager (version 5.4). Binary variables were expressed as risk ratios (RR) with 95% confidence intervals (CI), while continuous variables were expressed as mean differences (MD) with 95% CI. The meta-analysis model was selected based on heterogeneity testing results: a fixed-effect model was used when heterogeneity among studies was low (*I* ^*2*^ < 50% and *p* > 0.05); otherwise, a random-effects model was employed. Statistical significance was set at p < 0.05 (Cumpston et al., 2019).

### 2.7 Subgroup analysis and sensitivity analysis

To investigate heterogeneity among included studies, this research conducted predefined subgroup analyses to assess the impact of specific baseline factors on treatment outcomes. The predefined subgroups were: the concurrent radiotherapy and chemotherapy group and the chemotherapy-only group. Additionally, sensitivity analyses were performed using STATA 18.0 software by sequentially excluding individual studies to test the robustness of results and identify potential sources of heterogeneity.

### 2.8 Quality of evidence

The quality of evidence for each outcome measure was assessed and graded using the online tool GRADEpro (https://www.gradepro.org/) (Guyatt et al., 2008). The assessment covered five domains: risk of bias, inconsistency, indirectness, imprecision, and publication bias. Two researchers, YZ and DL, independently performed GRADE grading. Evidence grades were downgraded or upgraded based on predetermined criteria via footnotes, with detailed justifications provided for all decisions.

### 2.9 Trial sequential analysis (TSA)

When conducting a meta-analysis, minimizing the risk of erroneous conclusions due to random error is paramount. To achieve this, we further implemented TSA (Wetterslev et al., 2008). TSA employs an α-spending function, which establishes thresholds for statistical significance to control the risk of a type I error. It also applies a β-spending function and generates an invalidity boundary to control type II error. The Z-score is a statistic derived from the logarithm of the pooled intervention effect divided by its standard error. If the Z-curve crosses the power boundary or sequential monitoring boundary, we can conclude that the sample size is sufficient to detect the expected intervention effect, and further trials are unlikely to alter the study results (Wetterslev et al., 2017). Therefore, we used TSA software v.0.9.5.10 Beta (Copenhagen Trial Unit, Copenhagen, Denmark) to determine whether the required sample size should reach the threshold for statistical significance. The boundary type was set to two-sided, with a type I error rate of 5% and power of 0.80, and the relative risk reduction rate and control group event rate were set based on meta-analysis results (Wetterslev et al., 2017). We constructed cumulative Z-scores and required information sizes to firmly accept or reject the effect size of interest.

### 2.10 Publication bias

To assess potential publication bias, this study conducted a publication bias analysis for the primary outcomes (ORR and DCR). Funnel plots were generated using STATA 18.0 software for visual inspection, supplemented by Egger’s regression test to quantitatively evaluate funnel plot asymmetry. If evidence of publication bias was detected, the “trimming and filling method” was further applied to estimate the impact of bias on the pooled effect size.

## 3 Results

### 3.1 Search results and study characteristics

**FIGURE 1:**
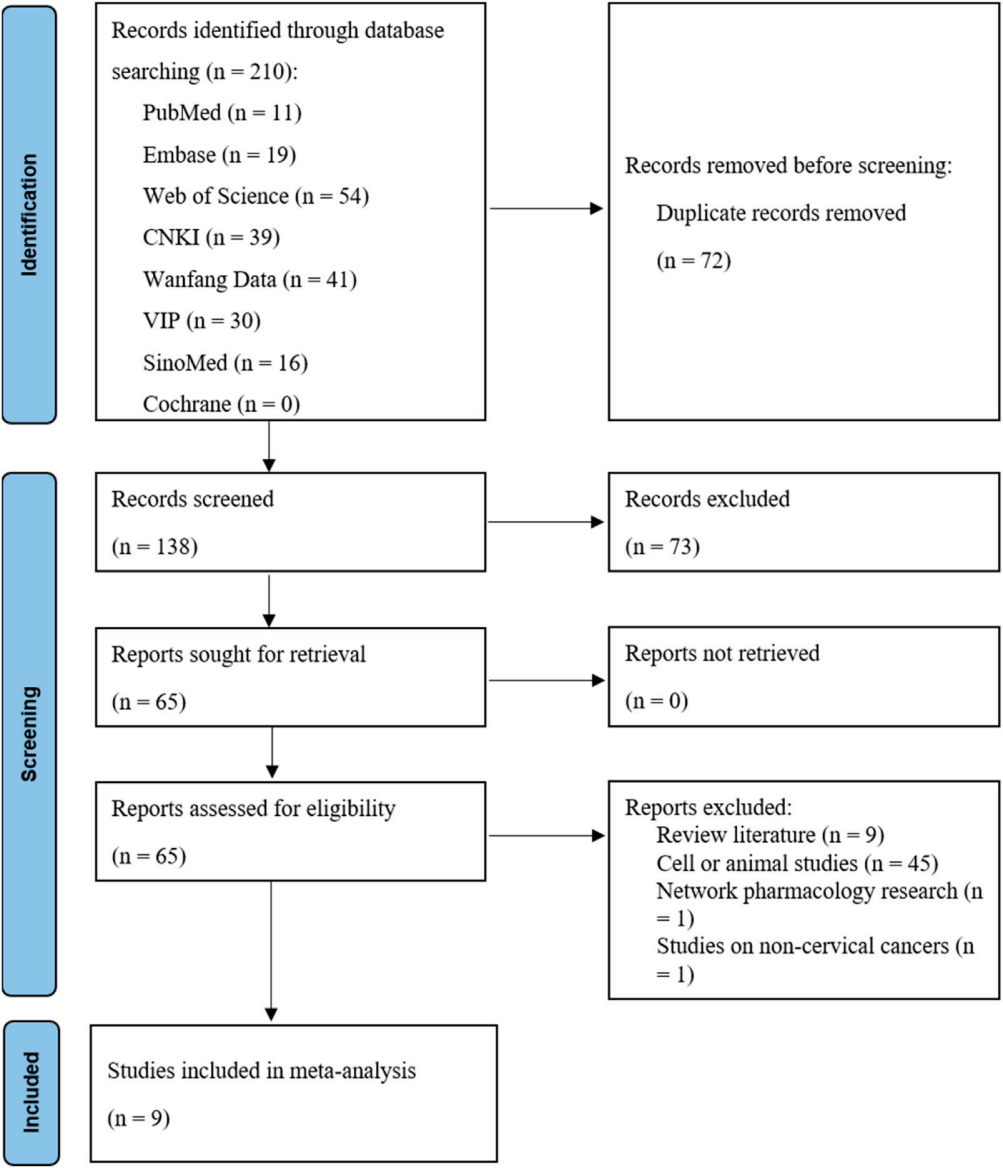
Flow diagram of the literature search.

The initial search yielded 210 relevant publications. After removing duplicates using EndNote 21.5 software, 138 unique studies remained. A preliminary screening based on titles and abstracts excluded 73 studies that did not meet the criteria. Subsequently, full-text reviews and assessments of the remaining 65 publications led to the exclusion of 56 studies. Ultimately, 9 studies met the inclusion criteria and were incorporated into the final analysis (Figure 1). All included studies were conducted in China, encompassing 776 patients (388 in each treatment and control group). They were published between 2012 and 2025, with sample sizes ranging from 40 to 130 patients. Regarding treatment regimens, four studies employed APS combined with concurrent chemoradiotherapy (TP regimen: paclitaxel + platinum-based agents), one study used APS combined with radiotherapy and cisplatin chemotherapy, two studies utilized APS combined with TP regimen chemotherapy, and two earlier studies employed APS combined with PVB chemotherapy (cisplatin + vincristine + bleomycin). Table 1 details the basic characteristics and treatment specifics of the included studies.

**TABLE 1:** The characteristics of all included studies.

Author and reference	Sample size		Mean age (years)		Interventions		Radiotherapy (total dose)	Chemotherapy cycles (number)	Outcomes
	T	C	T	C	T	C			
Ou (2012)	20	20	52.6		PVB + APS	PVB +rhG-CSF	-	Unknown	①②
Zhang (2014)	30	30	53.41 ± 5.03	52.21 ± 4.22	PVB + APS	PVB +rhG-CSF	-	Unknown	①②⑨
Li et al. (2016)	55	55	47.2 ± 4.0	48.4 ± 4.6	C + APS	R + TP	45 Gy	2	①②
Cui et al. (2018)	34	34	49.35 ± 13.51	48.82 ± 12.37	C + APS	TP	-	2	①②⑧⑩
Zhang et al. (2019)	50	50	51.2 ± 4.1	51.4 ± 3.8	C + APS	TP	-	8	①②③④⑤⑨⑩
Li et al. (2020b)	65	65	49.03 ± 4.62	46.97 ± 4.83	C + APS	R + TP	45 Gy IR: 10 Gy	2	①②③④⑤⑥⑨⑩
Xia et al. (2022)	50	50	50.80 ± 7.13	47.94 ± 9.33	C + APS	R + TP	45 Gy	4–6	③④⑤⑥⑦⑩
Su et al. (2025)	43	43	46.8 ± 2.5	46.2 ± 2.6	C + APS	R + TP	45 Gy IR: 6Gy	3–4	①②⑧⑩
Li et al. (2025c)	41	41	51.79 ± 2.80	51.82 ± 2.73	C + APS	R + Cisplatin	45–50.4Gy IR: 20–28Gy	3	①②④⑤⑥⑦⑩

Abbreviations: T, treatment group; C, control group; IR, intrauterine radiotherapy. Interventions: APS, Astragalus Polysaccharide Injection; PVB, Cisplatin + Vincristine + Bleomycin; TP, Paclitaxel + platinum-based agents (cisplatin/rupiramide/nedaplatin); rhG-CSF, Recombinant Human Granulocyte Colony-stimulating Factor Injection; R, radiation therapy; Outcomes: ①, ORR; ②, DCR; ③, CD3^+^ T lymphocyte ratio; ④, CD4^+^ T lymphocyte ratio; ⑤, CD8^+^ T lymphocyte ratio; ⑥, CD4^+^/CD8^+^ ratio; ⑦, Tumor marker levels (CEA, SCC, CA125); ⑧, Karnofsky Performance Status (KPS) score; ⑨, Blood cell counts: white blood cells, red blood cells, and platelets; ⑩, Incidence of adverse drug reactions (ADRs): gastrointestinal reactions, myelosuppression, and liver and kidney damage.

### 3.2 Risk of bias assessment

**FIGURE 2:**
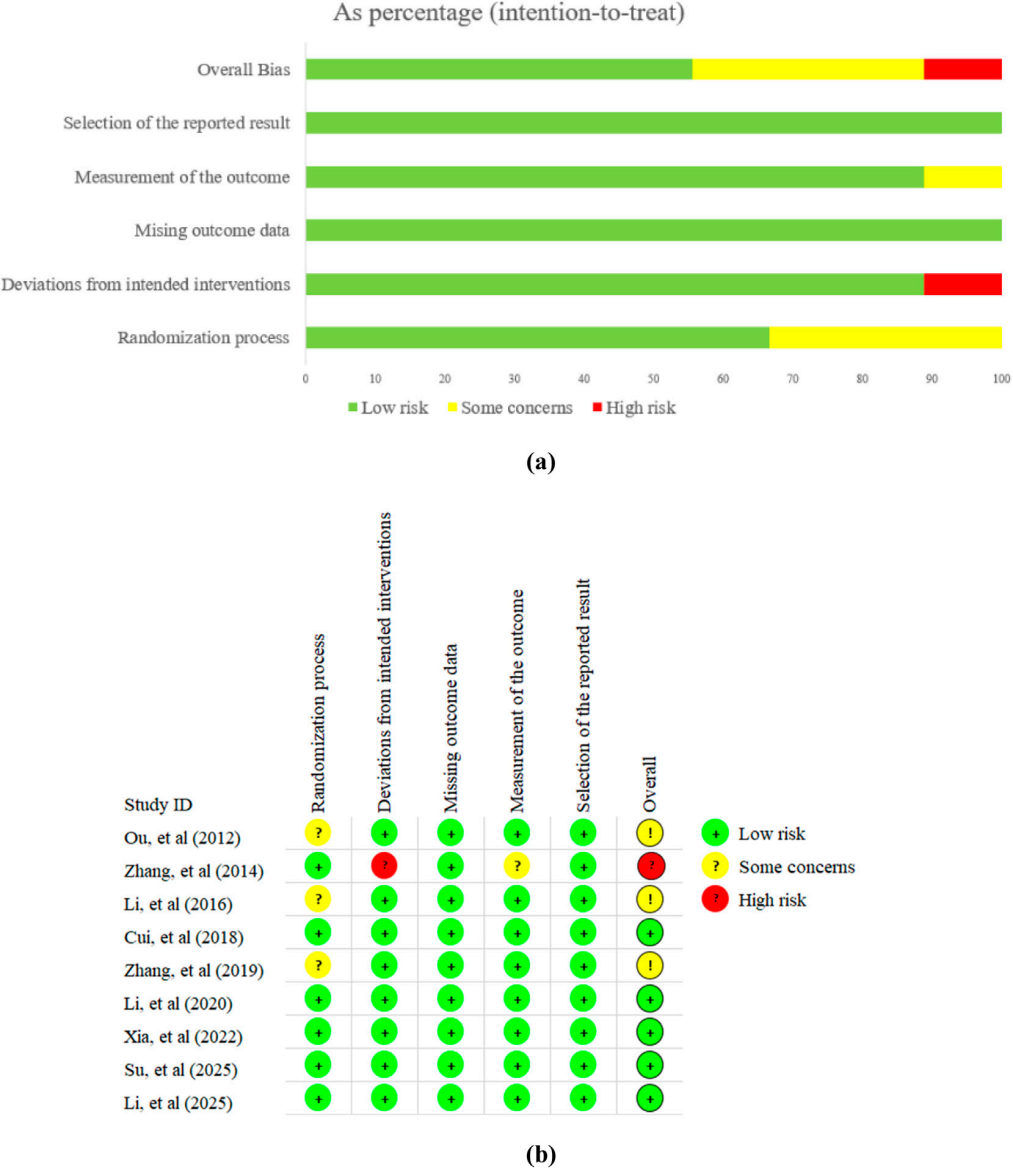
Risk of bias of included study. **(a)** risk of bias summary; **(b)** risk of bias graph.

In summary, 5 trials were considered to have a low risk of bias. Among the remaining 4 trials, 3 studies were assessed as “some concerns” due to the lack of clear randomization methods, while one study was assessed as “high risk” because it may have deviated from the established intervention. Detailed results of the risk of bias assessment are shown in Figure 2.

### 3.3 Primary outcomes

#### 3.3.1 ORR and DCR

**FIGURE 3:**
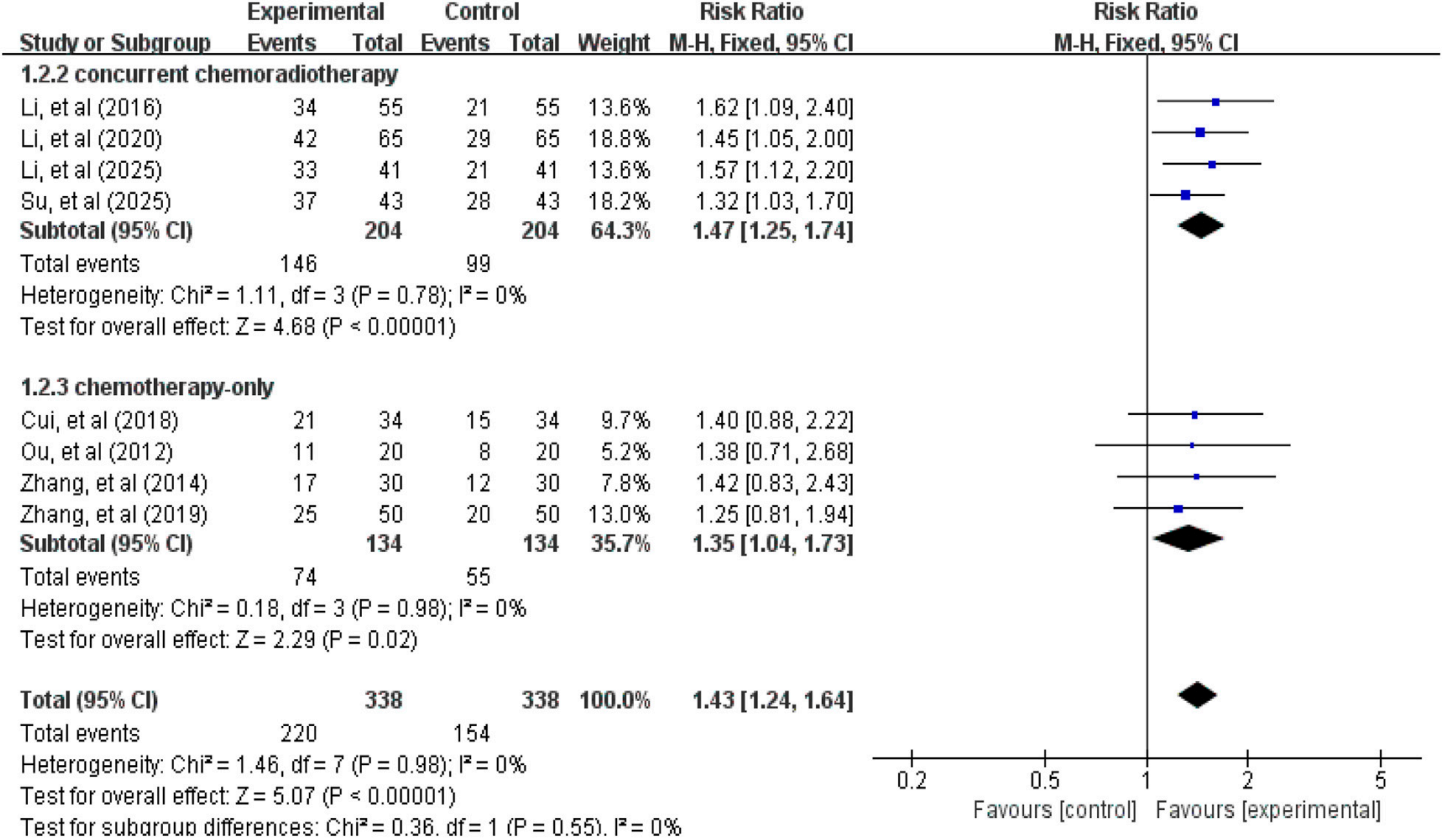
Forest plot of ORR.

**FIGURE 4:**
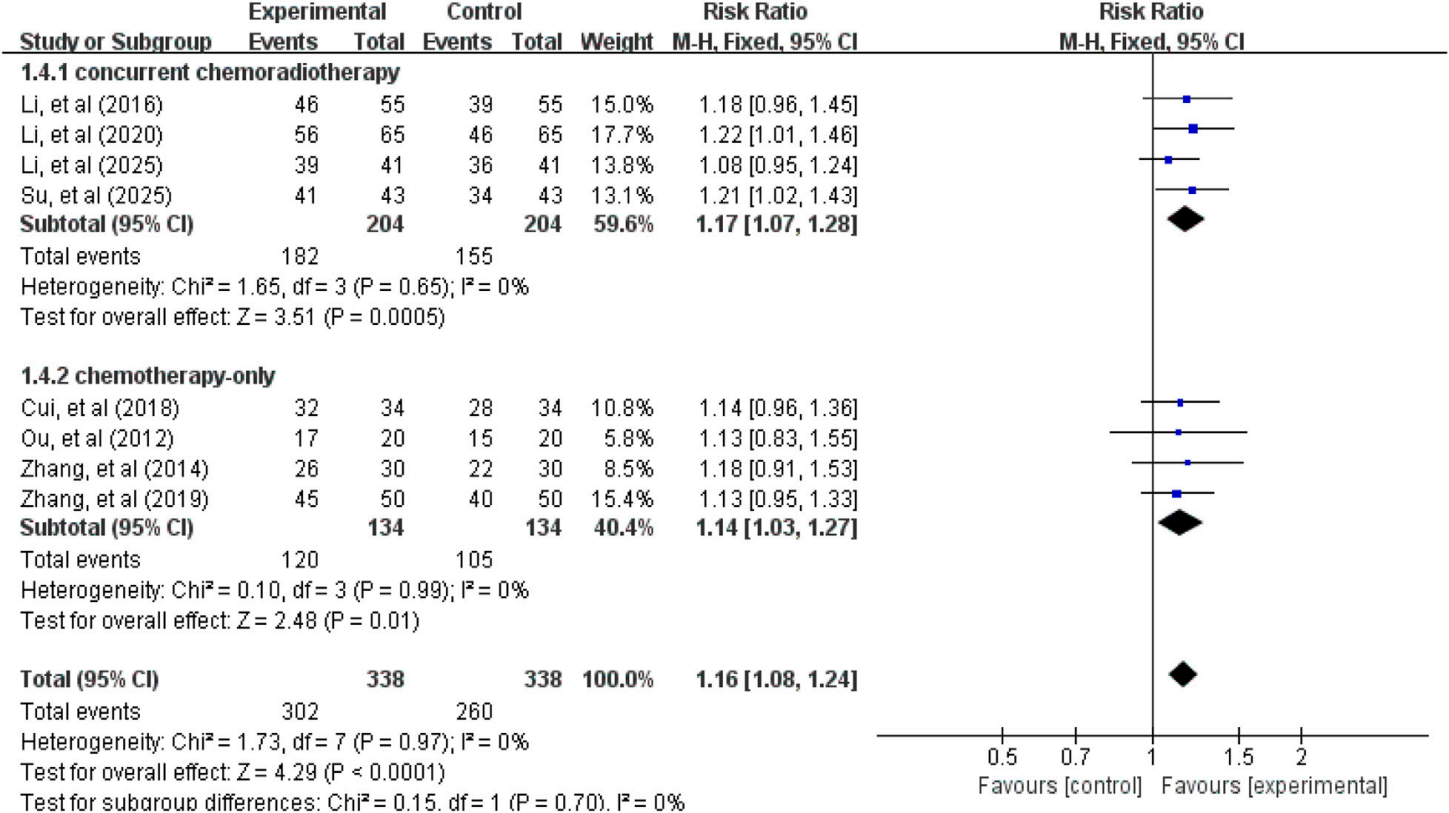
Forest plot of DCR.

A total of 8 studies (Ou, 2012; Zhang, 2014; Li et al., 2016; Cui et al., 2018; Zhang et al., 2019; Li et al., 2020b; Su et al., 2025; Li et al., 2025c) involving 676 patients reported ORR and DCR data. Tests for heterogeneity in ORR (χ^2^ = 1.46, *p* = 0.98, *I* ^*2*^ = 0%) and DCR (χ^2^ = 1.73, *p* = 0.97, *I* ^*2*^ = 0%) were not statistically significant, thus a fixed-effect model was used for effect size pooling. As shown in Figures 3, 4, the pooled analysis revealed that compared with the control group without APS combination therapy, the treatment group with APS combination therapy demonstrated a 43% increase in ORR (RR = 1.43, 95% CI: 1.24–1.64, *p* < 0.00001); and DCR increased by 16% (RR = 1.16, 95% CI: 1.08–1.24, *p* < 0.00001). To further investigate the efficacy differences of APS across treatment modalities, we conducted a subgroup analysis based on combination therapy regimens, distinguishing between the APS plus concurrent chemoradiotherapy group and the APS plus chemotherapy-only group. Results showed that compared with the control group, the APS plus concurrent chemoradiotherapy group (ORR: RR = 1.47, 95% CI: 1.25–1.74, *p* < 0.00001; DCR: RR = 1.17, 95% CI: 1.07–1.28, *p* = 0.0005) and the APS plus chemotherapy alone group (ORR: RR = 1.35, 95% CI: 1.04–1.73, *p* = 0.02; DCR: RR = 1.14, 95% CI: 1.03–1.27, *p* = 0.01). Notably, the improvement in efficacy was greater in the APS combined with concurrent chemoradiotherapy group than in the APS combined with chemotherapy alone group.

### 3.4 Secondary outcomes

#### 3.4.1 Immune indicators

**FIGURE 5:**
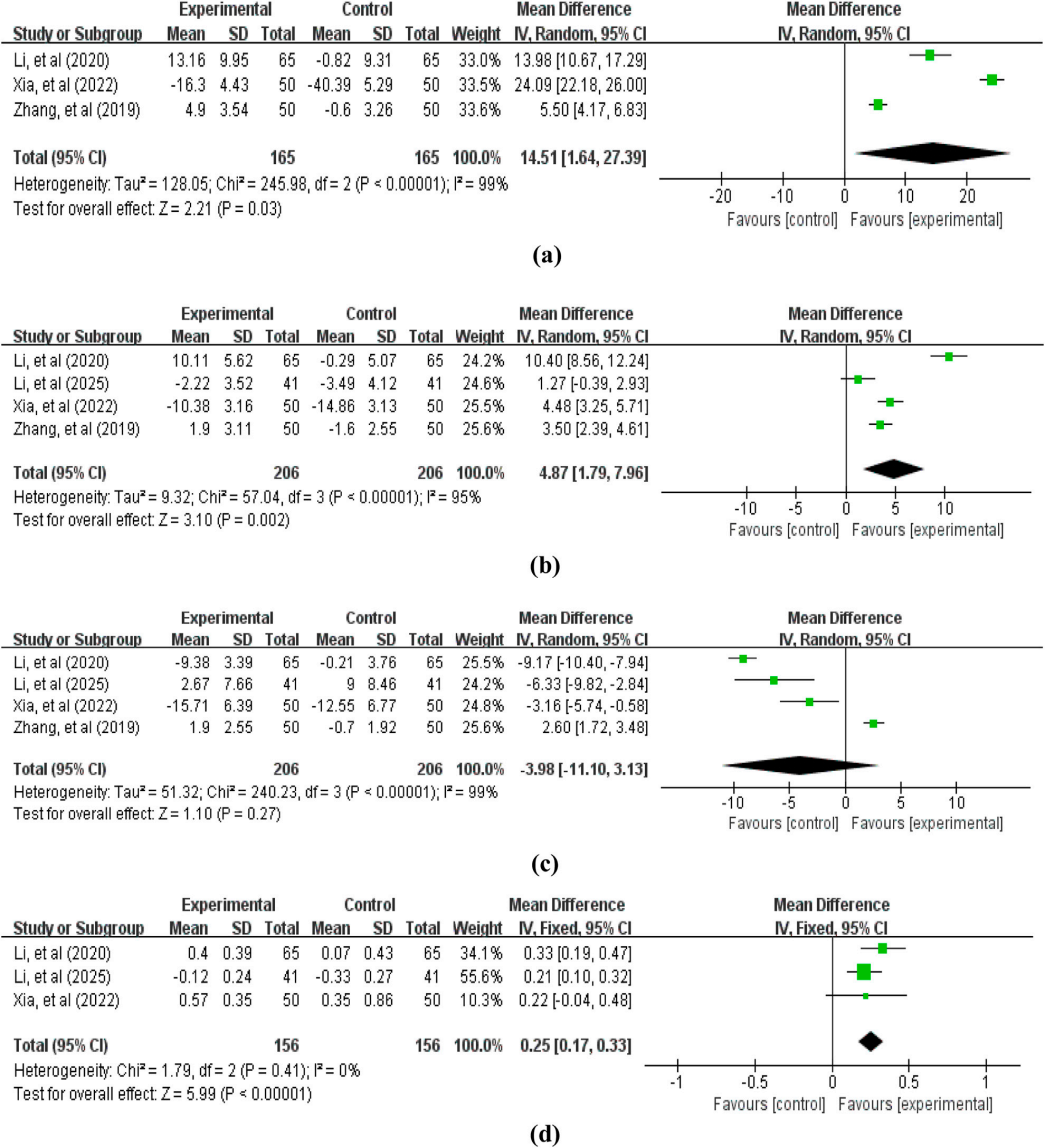
Forest plot of immune indicators: **(a)** CD3^+^ T lymphocyte ratio; **(b)** CD4^+^ T lymphocyte ratio; **(c)** CD8^+^ T lymphocyte ratio; **(d)** CD4^+^/CD8^+^ ratio.

Regarding immune function indicators, we primarily examined the CD3^+^ T lymphocyte ratio (3 studies (Zhang et al., 2019; Li et al., 2020b; Xia et al., 2022), 330 patients, Figure 5), CD4^+^ T lymphocyte ratio (4 studies (Zhang et al., 2019; Li et al., 2020b; Xia et al., 2022; Li et al., 2025c), 412 patients, Figure 5), CD8^+^ T lymphocyte ratio (4 studies (Zhang et al., 2019; Li et al., 2020b; Xia et al., 2022; Li et al., 2025c), 412 patients, Figure 5), and CD4^+^/CD8^+^ ratio (3 studies (Li et al., 2020b; Xia et al., 2022; Li et al., 2025c), 312 patients, Figure 5). The pooled analysis revealed that compared with the control group not receiving APS, the treatment group receiving APS significantly increased the CD3^+^ T lymphocyte ratio (MD = 14.51, 95% CI: 1.64–27.39, *p* = 0.03; heterogeneity: χ2 = 245.98, *p* < 0.00001, *I* ^*2*^ = 99%), the CD4^+^ T lymphocyte ratio (MD = 4.87, 95% CI: 1.79–7.96, *p* = 0.002; heterogeneity: χ2 = 57.04, *p* < 0.00001, *I* ^*2*^ = 95%), and the CD4^+^/CD8^+^ ratio (MD = 0.25, 95% CI: 0.17–0.33, *p* < 0.00001; heterogeneity: χ2 = 240.23, *p* < 0.00001, *I* ^*2*^ = 99%). However, the difference in CD8^+^ T lymphocyte ratio between the two groups was not statistically significant (MD = −3.98, 95% CI: -11.10–3.13, *p* = 0.27; heterogeneity: χ2 = 1.79, *p* = 0.41, *I* ^*2*^ = 0%).

#### 3.4.2 Tumor marker levels

**FIGURE 6:**
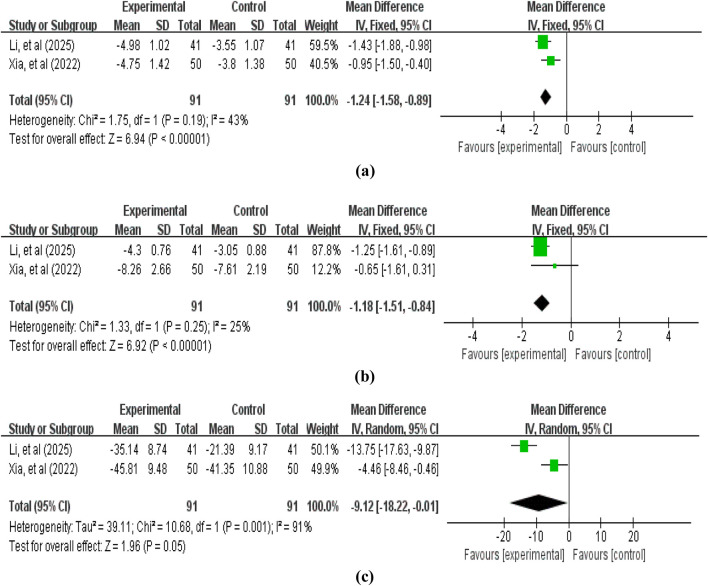
Forest plot of tumor marker levels: **(a)** carcinoembryonic antigen (CEA); **(b)** squamous cell carcinoma antigen (SCC); **(c)** carbohydrate antigen 125 (CA125).

2 studies (Xia et al., 2022; Li et al., 2025c) involving 182 patients reported tumor marker level data, including CEA, SCC, and CA125. The pooled analysis revealed that compared with the control group without APS, the treatment group with APS further reduced tumor marker levels: CEA (MD = −1.24, 95% CI: −1.58 to −0.89, *p* < 0.00001; heterogeneity: χ2 = 1.75, *p* = 0.19, *I* ^*2*^ = 43%), SCC (MD = −1.18, 95% CI: -1.51 to −0.84, *p* < 0.00001; heterogeneity: χ2 = 1.33, *p* = 0.25, *I* ^*2*^ = 25%), and CA125 (MD = −9.12, 95% CI: -18.22 to −0.01, *p* = 0.05; heterogeneity: χ2 = 10.68, *p* = 0.001, *I* ^*2*^ = 91%) (Figure 6).

#### 3.4.3 KPS score

**FIGURE 7:**
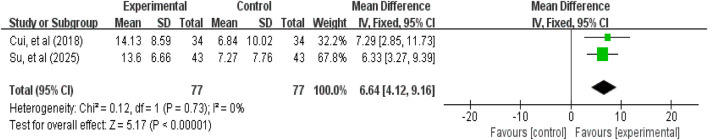
Forest plot of KPS.

2 studies (Cui et al., 2018; Su et al., 2025) involving 154 patients reported KPS score data. Heterogeneity testing revealed no significant heterogeneity (χ2 = 0.12, *p* = 0.73, *I* ^*2*^ = 0%), thus allowing the use of a fixed-effect model for combining effect sizes. As shown in Figure 7, the pooled analysis revealed that compared with the control group without APS, the treatment group with APS significantly improved patients’ KPS scores (MD = 6.64, 95% CI: 4.12–9.16, *p* < 0.00001). This suggests that APS contributes to further enhancing patients’ functional status and quality of life.

#### 3.4.4 Blood cell counts

**FIGURE 8:**
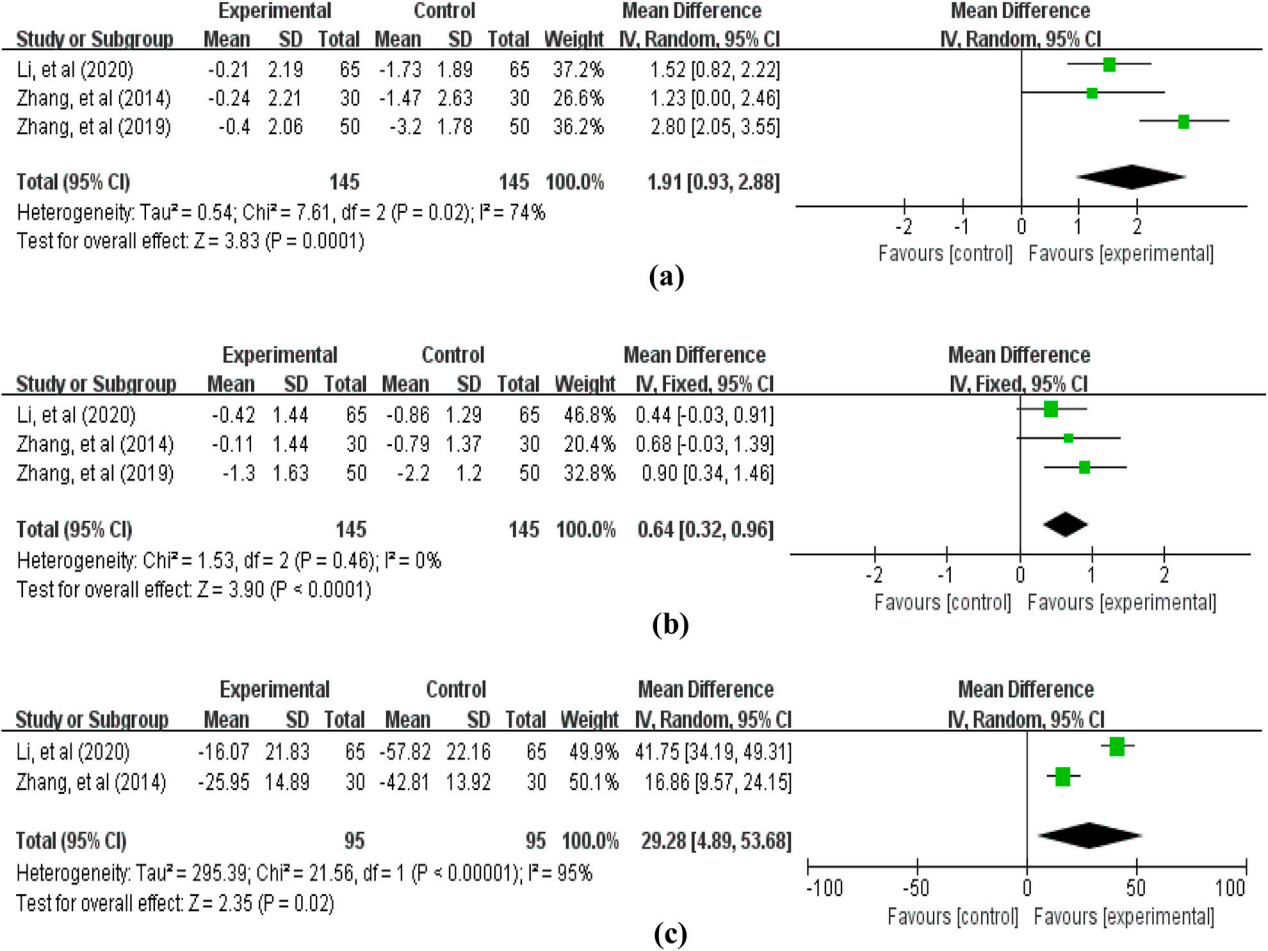
Forest plot of blood cell counts: **(a)** white blood cells; **(b)** red blood cells; **(c)** platelets.

The blood cell counts data reported in the included studies primarily encompassed: white blood cells (3 studies (Zhang, 2014; Zhang et al., 2019; Li et al., 2020b), involving 290 patients, Figure 8), red blood cells (3 studies (Zhang, 2014; Zhang et al., 2019; Li et al., 2020b), involving 290 patients, Figure 8), and platelets (2 studies (Zhang, 2014; Li et al., 2020b), involving 190 patients, Figure 8). The pooled analysis revealed that compared with the control group not receiving APS, the treatment group receiving APS significantly improved blood cell levels: white blood cells (MD = 1.91, 95% CI: 0.93–2.88, *p* = 0.0001; heterogeneity: χ2 = 7.61, *p* = 0.02, *I* ^*2*^ = 74%), red blood cells (MD = 0.64, 95% CI: 0.32–0.96, *p* < 0.0001; heterogeneity: χ2 = 1.53, *p* = 0.46, *I* ^*2*^ = 0%), and platelets (MD = 29.28, 95% CI: 4.89–53.68, *p* = 0.02; heterogeneity: χ2 = 21.56, *p* < 0.00001, *I* ^*2*^ = 95%).

#### 3.4.5 ADRs

**FIGURE 9:**
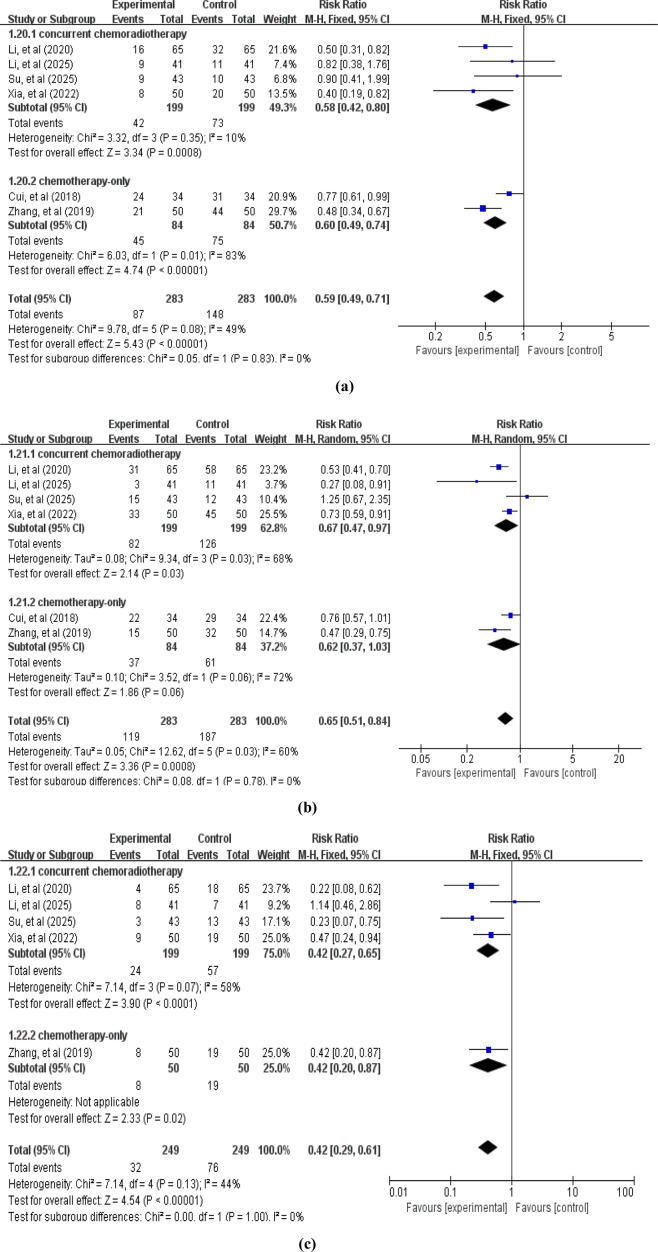
Forest plot of ADRs: **(a)** gastrointestinal reactions; **(b)** myelosuppression; **(c)** liver and kidney damage.

A total of 6 studies reported ADRs. Among these, primary outcomes included gastrointestinal reactions (6 studies (Cui et al., 2018; Zhang et al., 2019; Li et al., 2020b; Xia et al., 2022; Su et al., 2025; Li et al., 2025c), involving 566 patients, Figure 9), myelosuppression (6 studies (Cui et al., 2018; Zhang et al., 2019; Li et al., 2020b; Xia et al., 2022; Su et al., 2025; Li et al., 2025c), involving 566 patients, Figure 9), and liver and kidney damage (5 studies (Zhang et al., 2019; Li et al., 2020b; Xia et al., 2022; Su et al., 2025; Li et al., 2025c), involving 498 patients, Figure 9). The pooled analysis revealed that compared with the control group without APS combination therapy, the treatment group with APS combination therapy significantly reduced gastrointestinal reactions (RR = 0.59, 95% CI: 0.49–0.71, *p* < 0.00001; heterogeneity: χ2 = 9.78, *p* < 0.08, *I* ^*2*^ = 49%), myelosuppression (RR = 0.65, 95% CI: 0.51–0.84, *p* = 0.0008; heterogeneity: χ2 = 12.62, *p* = 0.03, *I* ^*2*^ = 60%), and liver and kidney damage (RR = 0.42, 95% CI: 0.29–0.61, *p* < 0.00001; heterogeneity: χ2 = 7.14, *p* = 0.13, *I* ^*2*^ = 44%).

### 3.5 Sensitivity analysis

**FIGURE 10:**
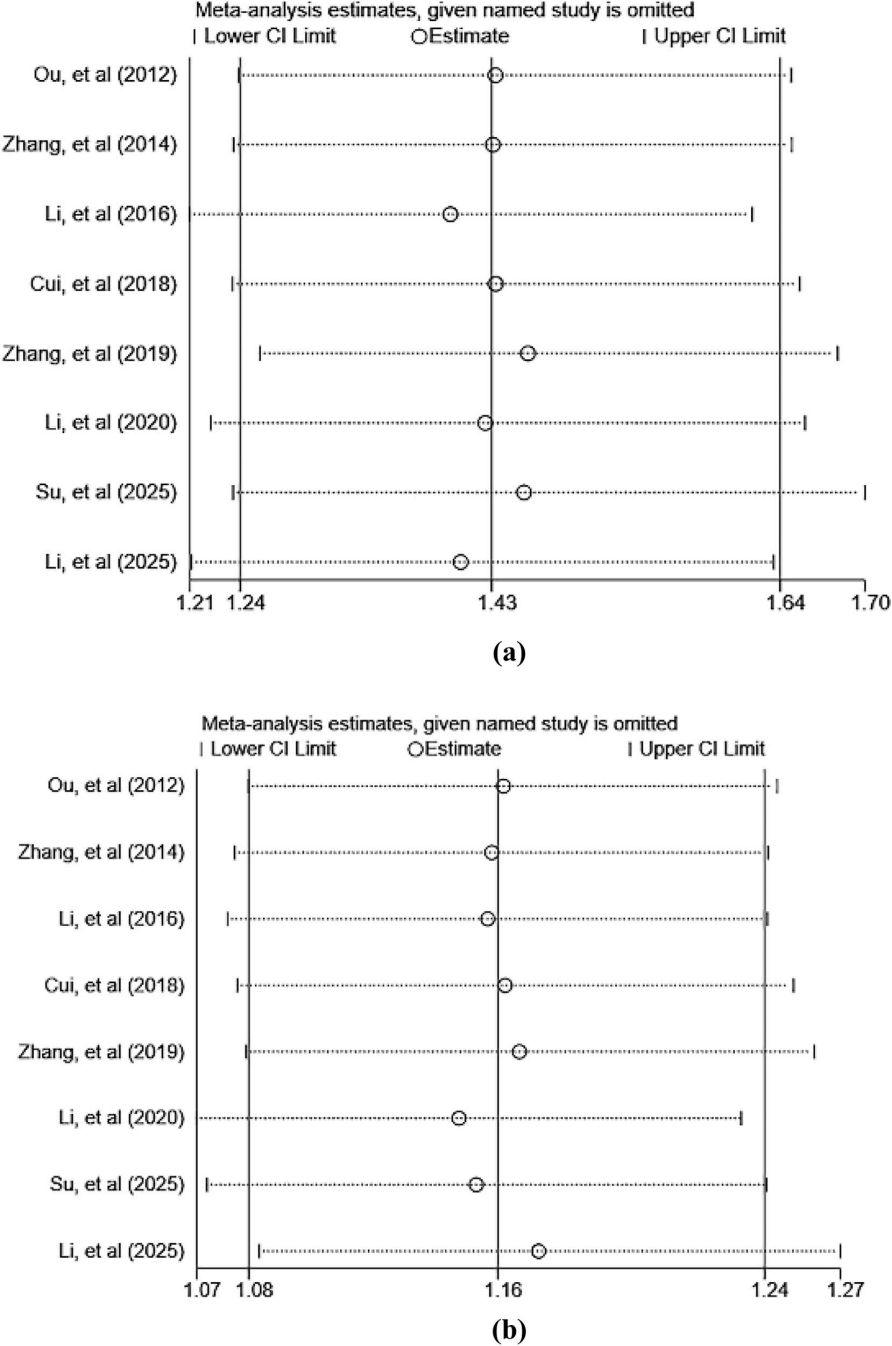
Sensitivity analyses of primary outcomes: **(a)** ORR; **(b)** DCR.

To assess the robustness and reliability of the primary outcome measures (ORR and DCR), this study employed a sensitivity analysis using the sequential exclusion method to evaluate the impact of each individual study on the overall pooled results. The analysis revealed that the pooled effect size did not undergo significant changes after sequentially excluding each study, indicating that the meta-analysis results are robust and reliable. The primary findings of the sensitivity analysis are presented in Figure 10. Detailed results of the sensitivity analysis for other outcomes are listed in Supplementary Material S3.

### 3.6 Quality of evidence

According to the evidence assessment of GRADEpro system, the quality of evidence for the two outcomes (ORR and DCR) was rated as low due to methodological limitations (lack of blinding and small sample size). Evidence quality for all other outcomes was rated as low or very low, primarily due to the following reasons: failure to address risk of bias from blinding, statistical heterogeneity, excessively wide confidence intervals, and limited number of studies. A summary of evidence grades for each outcome measure is provided in Supplementary Material S4.

### 3.7 Trial sequential analyses

**FIGURE 11:**
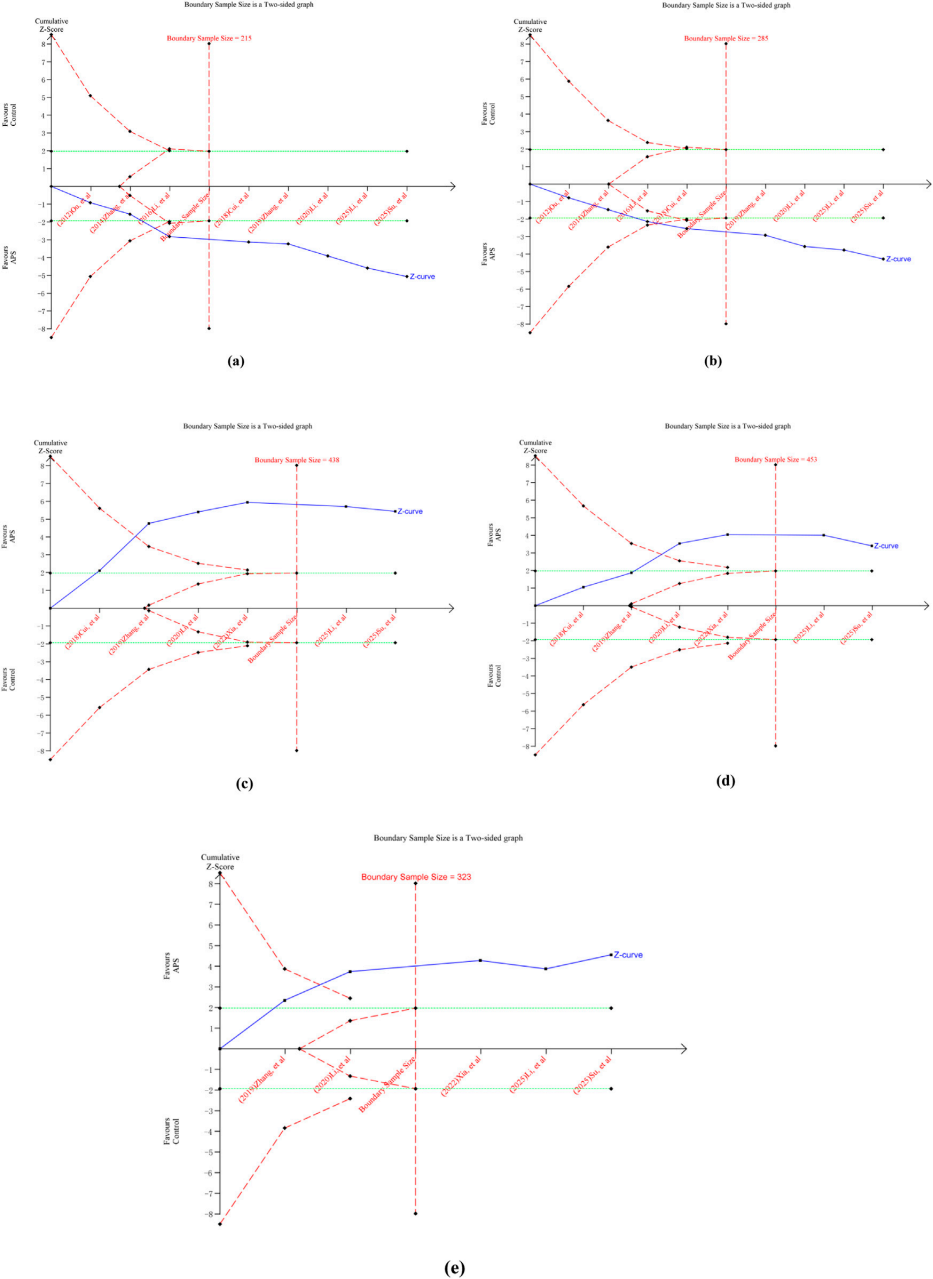
Outcomes of TSA. **(a)** ORR; **(b)** DCR; **(c)** gastrointestinal reactions; **(d)** myelosuppression; **(e)** liver and kidney damage.

For ORR, the cumulative Z-curve crossed both the conventional and trial sequential monitoring boundaries. This indicates a sample size of 215 patients is required to draw a stable conclusion (Figure 11a). For DCR, a sample size of 285 patients was determined to be necessary for a stable conclusion. The cumulative Z-curve for DCR also crossed both conventional and trial sequential monitoring boundaries (Figure 11b). Concurrently, separate TSA analysis for ADRs (gastrointestinal reactions, myelosuppression, liver and kidney damage) showed that the Z-curves for each crossed both the conventional and trial sequential monitoring boundaries (Figures 11c–e). Therefore, the study findings indicate that the current trials for ORR, DCR, and ADRs have achieved the required information volume. The results are both robust and reliable, suggesting that additional trials are unlikely to alter the established conclusions.

### 3.8 Publication bias

**FIGURE 12:**
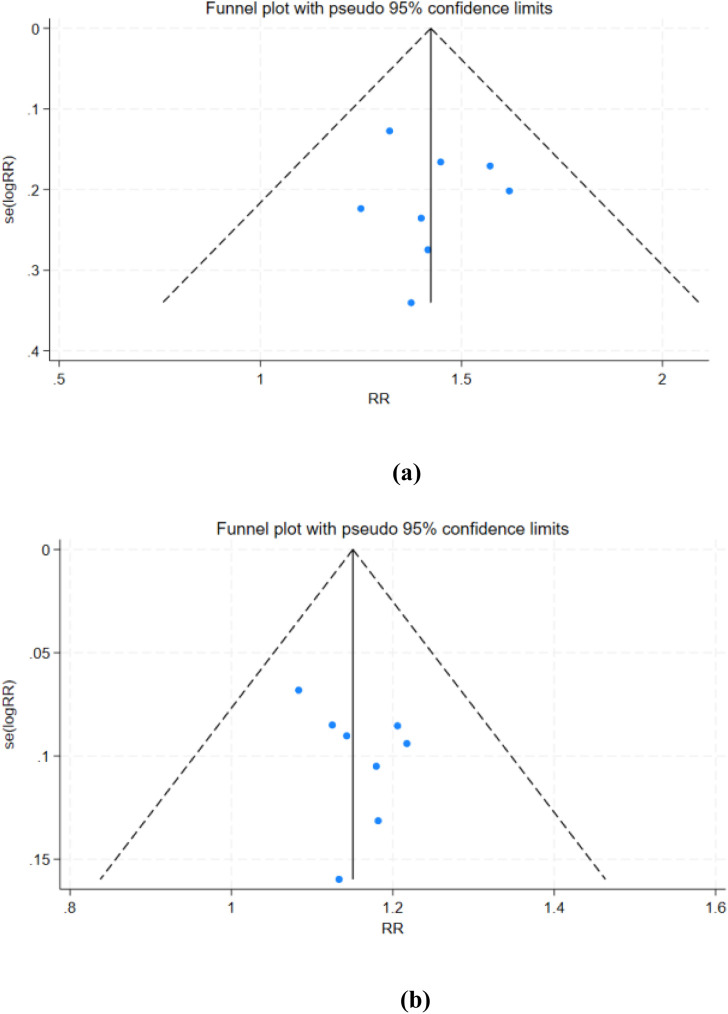
Funnel plots of ORR and DCR. **(a)** ORR; **(b)** DCR.

For the primary efficacy endpoints ORR and DCR, we conducted a comprehensive assessment of publication bias using funnel plots and Egger’s test. The funnel plots for ORR (Figure 12a) and DCR (Figure 12b) showed no significant asymmetry, and Egger’s test results (ORR: p = 0.830; DCR: p = 0.302) further confirmed the absence of substantial publication bias.

## 4 Discussion

### 4.1 Principal findings

We retrieved as many RCTs as possible and conducted a meta-analysis. Results indicate that APS, when used as an adjunct therapy alongside radiotherapy and chemotherapy for cervical cancer, appears to improve short-term efficacy—a finding validated by the TSA. Subgroup analyses further suggest that APS may enhance the efficacy of radiotherapy or chemotherapy independently. Additionally, APS combination therapy is associated with improved physical performance status, immune function regulation, reduced tumor marker levels, and mitigated adverse effects of radiotherapy and chemotherapy. These findings suggest its potential clinical application value.

Recent evidence indicates that APS enhances the efficacy of chemotherapy and radiotherapy in cervical cancer by modulating key signaling pathways and immune responses. Specifically, APS inhibits the cisplatin resistance pathway and regulates the cell cycle by suppressing the Wnt/β-catenin pathway via the PPARD/CDC20 axis (Liu et al., 2025); APS also influences autophagy and upregulates γH2AX expression, thereby enhancing cervical cancer sensitivity to radiotherapy (Zhai et al., 2018; Liu et al., 2024). *In vivo* studies further validate these mechanisms: APS alleviates endoplasmic reticulum stress and promotes mitochondrial autophagy, thereby enhancing apoptosis and mitigating cisplatin-induced toxicity (Chen et al., 2025). Consistent with these findings, our meta-analysis suggests the potential role of APS as an adjuvant therapy in cervical cancer treatment. Furthermore, our study revealed that APS appears to modulate key immune parameters in cervical cancer patients, including the ratio of CD3^+^ and CD4^+^ T cells and the CD4^+^/CD8^+^ ratio, corroborating its well-established potent immunomodulatory properties (Liu et al., 2021; Yu et al., 2021; Li et al., 2022, Bai et al., 2025). Collectively, these mechanisms provide a rational basis for considering APS as an adjuvant therapy for cervical cancer.

### 4.2 Strengths and comparison with other studies

Our findings align with previous meta-analyses evaluating APS as an adjunctive therapy for various malignancies including lung, gastric, liver, and esophageal cancers, which suggested that APS combination therapy improves short-term clinical efficacy, quality of life, and immune function (Yang et al., 2021; Zhang et al., 2020; Ge et al., 2015; Miao et al., 2019; Shi et al., 2016). However, high-quality evidence-based data supporting the efficacy and safety of APS combined with chemoradiotherapy for cervical cancer remains lacking. Therefore, this study conducted a systematic review and meta-analysis based on the latest clinical evidence, demonstrating several advantages over previous reviews: (1) Subgroup analysis revealed that APS improved ORR and DCR in cervical cancer patients receiving either chemotherapy alone or concurrent chemoradiotherapy, with more pronounced benefits observed in the concurrent chemoradiotherapy group. This further supports the synergistic enhancement effect of APS on radiotherapy and chemotherapy, respectively; (2) Multidimensional validation of APS’s adjuvant value in enhancing efficacy and reducing toxicity during cervical cancer treatment was achieved by analyzing its effects on immune function indicators, KPS scores, blood cell counts, and ADRs incidence; (3) Sensitivity analyses and evidence grading based on the GRADE system enhanced the reliability of pooled results. (4) TSA indicated that existing evidence has reached sufficient information size for key endpoints (ORR, DCR, and ADRs). This study provides more reliable and cancer-specific evidence supporting the incorporation of APS into adjuvant treatment regimens for cervical cancer radiotherapy and chemotherapy.

### 4.3 Limitations

However, this study has several limitations. First, despite systematically searching authoritative domestic and international databases and manually reviewing relevant literature, only 9 studies meeting inclusion criteria were identified after rigorous screening, involving a total of 776 patients. The small number of included studies and limited sample sizes (ranging from 40 to 130 participants, with a mean of 86 participants per trial) resulted in insufficient data for pooled analyses of certain outcome measures. This occasionally led to unexplained heterogeneity, further weakening the strength of the evidence. Second, all included RCTs were conducted in China, limiting the generalizability of our findings to populations in other regions. Additionally, due to the small-study effects and single-country evidence, the possibility of publication bias cannot be ruled out. Third, the methodological quality of the included RCTs was generally low. 6 trials reported using “random number tables” or “computer-generated randomization,” while the remaining 3 trials merely mentioned “randomization” without providing further details. None of the included RCTs reported allocation concealment or blinding implementation. These deficiencies may increase the risk of implementation and measurement bias, particularly affecting the reliability of subjective outcome measures such as KPS score. Finally, as most studies lacked long-term follow-up data, this review could not assess the long-term efficacy of APS combined with chemoradiotherapy for cervical cancer nor evaluate whether this therapy yields sustained clinical benefits.

### 4.4 Implications for research and clinical practice

Given these limitations, future research should address the urgent need for larger-scale, multicenter RCTs employing rigorous prospective designs to ensure reliable outcomes. To enhance the generalizability of findings, studies should incorporate more diverse global populations to support the development of more effective public health strategies. With the rapid advancement of immunotherapy and targeted therapy in cervical cancer treatment, future trials should include long-term survival endpoints (e.g., PFS, OS) and validated quality-of-life measures, and evaluate APS with contemporary treatments such as immunotherapy. Furthermore, additional RCTs are needed to validate whether APS efficacy exhibits time-dependent and concentration-dependent effects through pre-specified subgroup analyses. These trials should also compare APS efficacy across different clinical stages of cervical cancer and when combined with various chemoradiotherapy regimens.

## 5 Conclusion

In summary, the results of this systematic review and meta-analysis of 9 RCTs (involving 776 cervical cancer patients) indicate that APS combined with radiochemotherapy may improve short-term efficacy and reduce toxicity in cervical cancer, but further large, rigorously designed multicenter RCTs are required to confirm these benefits.

## Data Availability

The original contributions presented in the study are included in the article/Supplementary Material, further inquiries can be directed to the corresponding authors.
