# Supplementary material for: Astragalus polysaccharides combined with radiochemotherapy for cervical cancer: a systematic review and meta-analysis of randomized controlled studies
Source: Front Pharmacol. 2025 Nov 11;16:1699902. doi: 10.3389/fphar.2025.1699902 (PMC12643993; doi:10.3389/fphar.2025.1699902)
Supplement: Supplementary file 1 [file Supplementaryfile1.docx]

Astragalus Polysaccharides Combined with Radiochemotherapy for Cervical Cancer: A Systematic Review and Meta-analysis of Randomized Controlled Studies

Search formula：

CNKI：39 articles

（主题：黄芪多糖 + APS + API + 黄芪多糖注射液 + 黄芪多糖粉 + 黄芪多糖溶液 + 黄芪多糖提取 + 黄芪多糖口服液 + 黄芪多糖脂质体 + 黄芪多糖粉针剂 + 黄芪多糖冲剂 + 注射用黄芪多糖）

AND

（主题：宫颈癌 + 宫颈恶性肿瘤 + 子宫颈癌 + 子宫颈恶性肿瘤 + 宫颈肿瘤 + 子宫颈肿瘤 + 宫颈鳞癌 + 宫颈鳞状细胞癌 + 子宫颈鳞癌 + 子宫颈鳞状细胞癌 + 宫颈腺癌 + 子宫颈腺癌 + 宫颈腺鳞癌 + 子宫颈腺鳞癌 + 宫颈神经内分泌癌 + 子宫颈神经内分泌癌）

WangFang：41 articles

（主题：黄芪多糖 OR APS OR 黄芪多糖注射液 OR 黄芪多糖粉 OR 黄芪多糖溶液 OR 黄芪多糖提取 OR 黄芪多糖口服液 OR 黄芪多糖脂质体 OR 黄芪多糖粉针剂 OR 黄芪多糖冲剂 OR 注射用黄芪多糖）

AND

（主题：宫颈癌 OR 宫颈恶性肿瘤 OR 子宫颈癌 OR 子宫颈恶性肿瘤 OR 宫颈肿瘤 OR 子宫颈肿瘤 OR 宫颈鳞癌 OR 宫颈鳞状细胞癌 OR 子宫颈鳞癌 OR 子宫颈鳞状细胞癌 OR 宫颈腺癌 OR 子宫颈腺癌 OR 宫颈腺鳞癌 OR 子宫颈腺鳞癌 OR 宫颈神经内分泌癌 OR 子宫颈神经内分泌癌）

VIP：30 articles

（题名或关键词：黄芪多糖 OR APS OR 黄芪多糖注射液 OR 黄芪多糖粉 OR 黄芪多糖溶液 OR 黄芪多糖提取 OR 黄芪多糖口服液 OR 黄芪多糖脂质体 OR 黄芪多糖粉针剂 OR 黄芪多糖冲剂 OR 注射用黄芪多糖）

AND

（题名或关键词：宫颈癌 OR 宫颈恶性肿瘤 OR 子宫颈癌 OR 子宫颈恶性肿瘤 OR 宫颈肿瘤 OR 子宫颈肿瘤 OR 宫颈鳞癌 OR 宫颈鳞状细胞癌 OR 子宫颈鳞癌 OR 子宫颈鳞状细胞癌 OR 宫颈腺癌 OR 子宫颈腺癌 OR 宫颈腺鳞癌 OR 子宫颈腺鳞癌 OR 宫颈神经内分泌癌 OR 子宫颈神经内分泌癌）

Sinomed：16 articles

( "黄芪多糖"[常用字段:智能] OR "APS"[常用字段:智能] OR "黄芪多糖注射液"[常用字段:智能] OR "黄芪多糖粉"[常用字段:智能] OR "黄芪多糖溶液"[常用字段:智能] OR "黄芪多糖提取"[常用字段:智能] OR "黄芪多糖口服液"[常用字段:智能] OR "黄芪多糖脂质体"[常用字段:智能] OR "黄芪多糖粉针剂"[常用字段:智能] OR "黄芪多糖冲剂"[常用字段:智能] OR "注射用黄芪多糖"[常用字段:智能])

AND

( "宫颈癌"[常用字段:智能] OR "宫颈恶性肿瘤"[常用字段:智能] OR "子宫颈癌"[常用字段:智能] OR "子宫颈恶性肿瘤"[常用字段:智能] OR "宫颈肿瘤"[常用字段:智能] OR "子宫颈肿瘤"[常用字段:智能] OR "宫颈鳞癌"[常用字段:智能] OR "宫颈鳞状细胞癌"[常用字段:智能] OR "子宫颈鳞癌"[常用字段:智能] OR "子宫颈鳞状细胞癌"[常用字段:智能] OR "宫颈腺癌"[常用字段:智能] OR "子宫颈腺癌"[常用字段:智能] OR "宫颈腺鳞癌"[常用字段:智能] OR "子宫颈腺鳞癌"[常用字段:智能] OR "宫颈神经内分泌癌"[常用字段:智能] OR "子宫颈神经内分泌癌"[常用字段:智能])

Pubmed：11 articles

(astragalus polysaccharides[MeSH Terms] OR APS[Title/Abstract] OR PG2[Title/Abstract] OR astragalus polysaccharide injection[Title/Abstract] OR astragalus polysaccharide for injection[Title/Abstract])

AND

(Cervical Cancer[MeSH Terms] OR Neoplasm, Uterine Cervical[Title/Abstract] OR Uterine Cervical Neoplasm[Title/Abstract] OR Neoplasms, Cervix[Title/Abstract] OR Cervix Neoplasm[Title/Abstract] OR Neoplasm, Cervix[Title/Abstract] OR Cervix Neoplasms[Title/Abstract] OR Cervical Neoplasms[Title/Abstract] OR Cervical Neoplasm[Title/Abstract] OR Neoplasms, Cervical[Title/Abstract] OR Cancer of the Uterine Cervix[Title/Abstract] OR Cancer of Cervix[Title/Abstract] OR Cancer of the Cervix[Title/Abstract] OR Cervix Cancer[Title/Abstract] OR Cancer, Cervix[Title/Abstract] OR Uterine Cervical Cancer[Title/Abstract] OR Cancer, Uterine Cervical[Title/Abstract] OR Cervical Cancer, Uterine[Title/Abstract] OR Uterine Cervical Cancers[Title/Abstract] OR Cervical Neoplasm, Uterine[Title/Abstract] OR Cancer, Cervical[Title/Abstract] OR Cervical Cancers[Title/Abstract])

Web of science：54 articles

astragalus polysaccharides OR APS OR PG2 OR astragalus polysaccharide injection OR astragalus polysaccharide for injection (Topic)

AND

Cervix Cancer OR Neoplasm, Uterine Cervical OR Uterine Cervical Neoplasm OR Neoplasms, Cervix OR Cervix Neoplasm OR Neoplasm, Cervix OR Cervix Neoplasms OR Cervical Neoplasms OR Cervical Neoplasm OR Neoplasms, Cervical OR Cancer of the Uterine Cervix OR Cancer of Cervix OR Cancer of the Cervix OR Cervical Neoplasm, Uterine OR Cancer, Cervix OR Uterine Cervical Cancer OR Cancer, Uterine Cervical OR Cervical Cancer, Uterine OR Uterine Cervical Cancers OR Cervical Cancer OR Cancer, Cervical OR Cervical Cancers (Topic)

Embase：19 articles

('astragalus polysaccharides':ti,ab,kw OR 'astragalus polysaccharide':ti,ab,kw OR 'APS':ti,ab,kw OR 'PG2':ti,ab,kw OR 'astragalus polysaccharide injection':ti,ab,kw OR 'astragalus polysaccharide for injection':ti,ab,kw)

AND

('Ca cervix':ti,ab,kw OR 'cancer of the cervix':ti,ab,kw OR 'cancer of the cervix uteri':ti,ab,kw OR 'cancer of the uterine cervix':ti,ab,kw OR 'cancer, uterine cervix':ti,ab,kw OR 'carcinogenesis of the cervix':ti,ab,kw OR 'cervical cancer':ti,ab,kw OR 'cervical cancerogenesis':ti,ab,kw OR 'cervical carcinogenesis':ti,ab,kw OR 'cervical malignancies':ti,ab,kw OR 'cervical malignancy':ti,ab,kw OR 'cervix ca':ti,ab,kw OR 'cervix cancer':ti,ab,kw OR 'cervix cancer, recurrent':ti,ab,kw OR 'cervix cancer, uterine':ti,ab,kw OR 'cervix cancerogenesis':ti,ab,kw OR 'cervix carcinogenesis':ti,ab,kw OR 'cervix malignancies':ti,ab,kw OR 'cervix malignancy':ti,ab,kw OR 'cervix malignancy, recurrent':ti,ab,kw OR 'cervix uteri cancer':ti,ab,kw OR 'cervix uterus cancer':ti,ab,kw OR 'malignancies of the cervix':ti,ab,kw OR 'malignancy of the cervix':ti,ab,kw OR 'neoplasma cervicis recurrens':ti,ab,kw OR 'neoplasma cervicis uteri recurrens':ti,ab,kw OR 'recurrent cancer of the cervix':ti,ab,kw OR 'recurrent cervix cancer':ti,ab,kw OR 'recurrent cervix malignancy':ti,ab,kw OR 'uterine cervical cancer':ti,ab,kw OR 'uterine cervix cancer, recurrent':ti,ab,kw OR 'uterine cervix malignancy, recurrent':ti,ab,kw OR 'uterine neck cancer':ti,ab,kw OR 'uterus cervix cancer':ti,ab,kw OR 'uterine cervix cancer':ti,ab,kw)

Cochrane：0 articles

(astragalus polysaccharides OR APS OR PG2 OR astragalus polysaccharide injection OR astragalus polysaccharide for injection in Title Abstract Keyword

AND

Neoplasm, Uterine Cervical; Neoplasms, Cervical; Cervix Neoplasms; Cervical Neoplasm; Cervix Neoplasm; Cervical Neoplasms; Cervical Neoplasm, Uterine; Neoplasms, Cervix; Uterine Cervical Neoplasm; Neoplasm, Cervix; Cervical Cancers; Cervix Cancer; Cancer, Cervical; Uterine Cervical Cancers; Cervical Cancer, Uterine; Cancer of Cervix; Uterine Cervical Cancer; Cancer, Uterine Cervical; Cancer of the Cervix; Cancer of the Uterine Cervix; Cervical Cancer; Cancer, Cervix in Title Abstract Keyword)
