# Supplementary material for: Astragalus polysaccharides combined with radiochemotherapy for cervical cancer: a systematic review and meta-analysis of randomized controlled studies
Source: Front Pharmacol. 2025 Nov 11;16:1699902. doi: 10.3389/fphar.2025.1699902 (PMC12643993; doi:10.3389/fphar.2025.1699902)
Supplement: Supplementary file 2 [file Supplementaryfile3.docx]

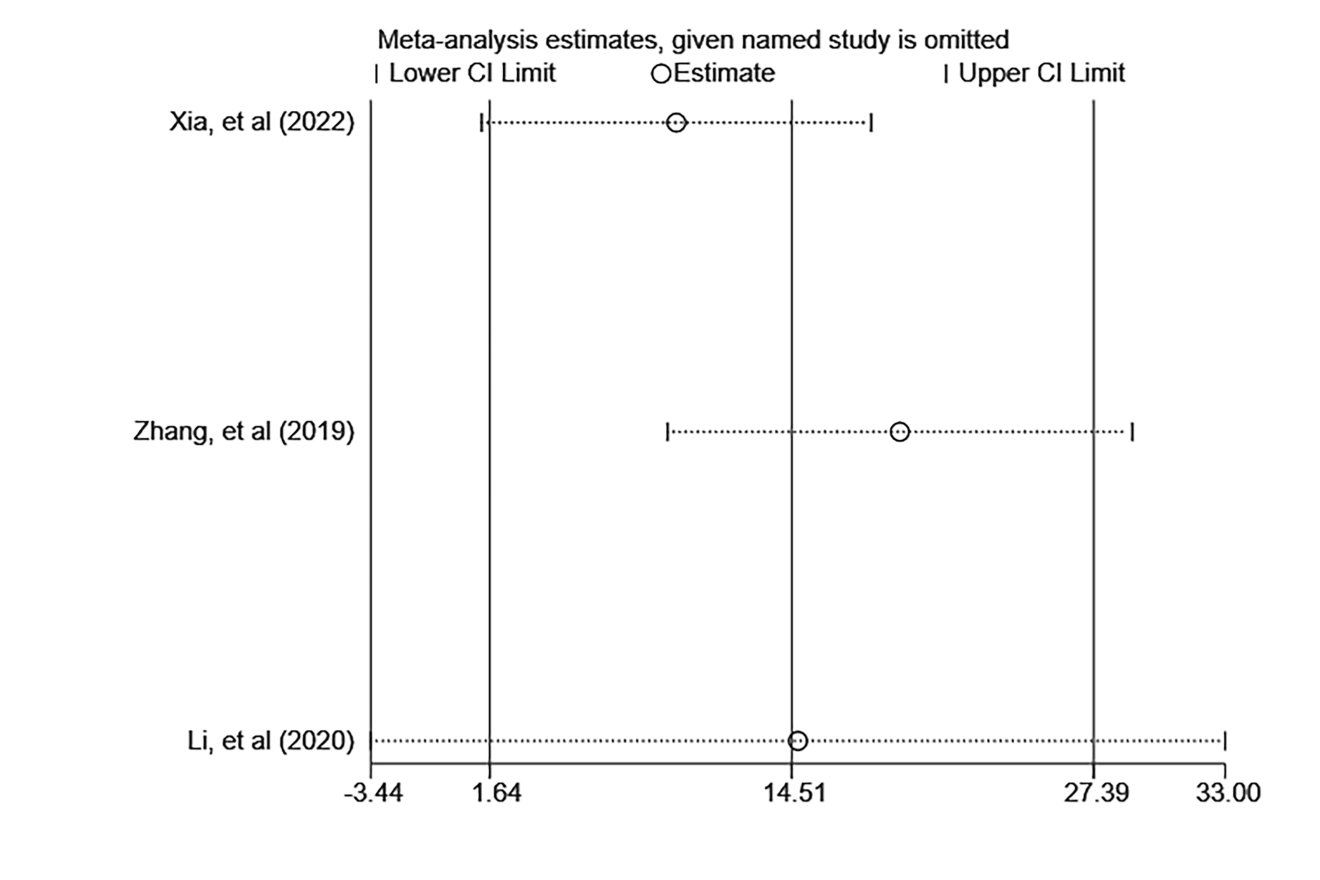


Sensitivity analyses of CD3⁺ T lymphocyte ratio


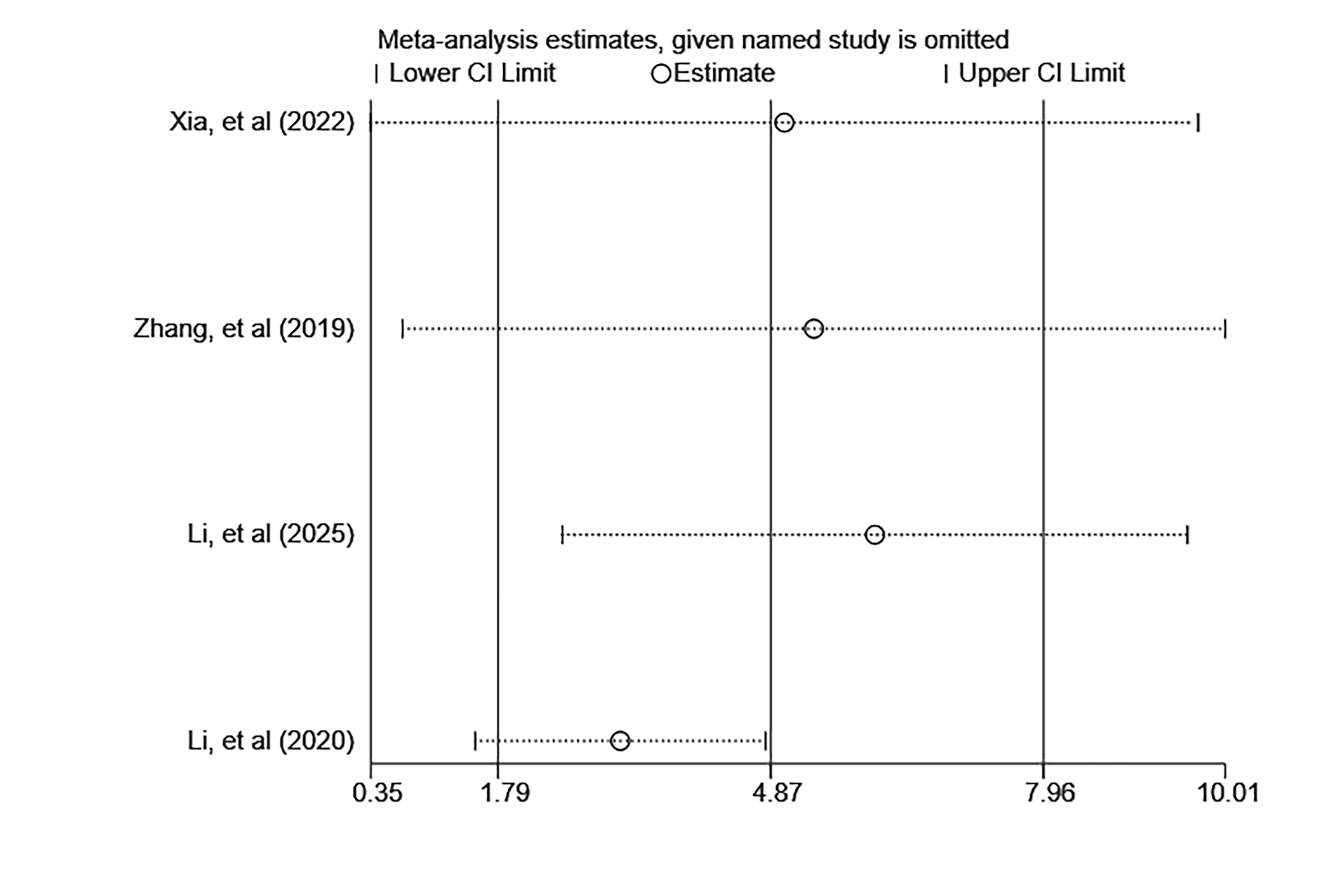


Sensitivity analyses of CD4⁺ T lymphocyte ratio


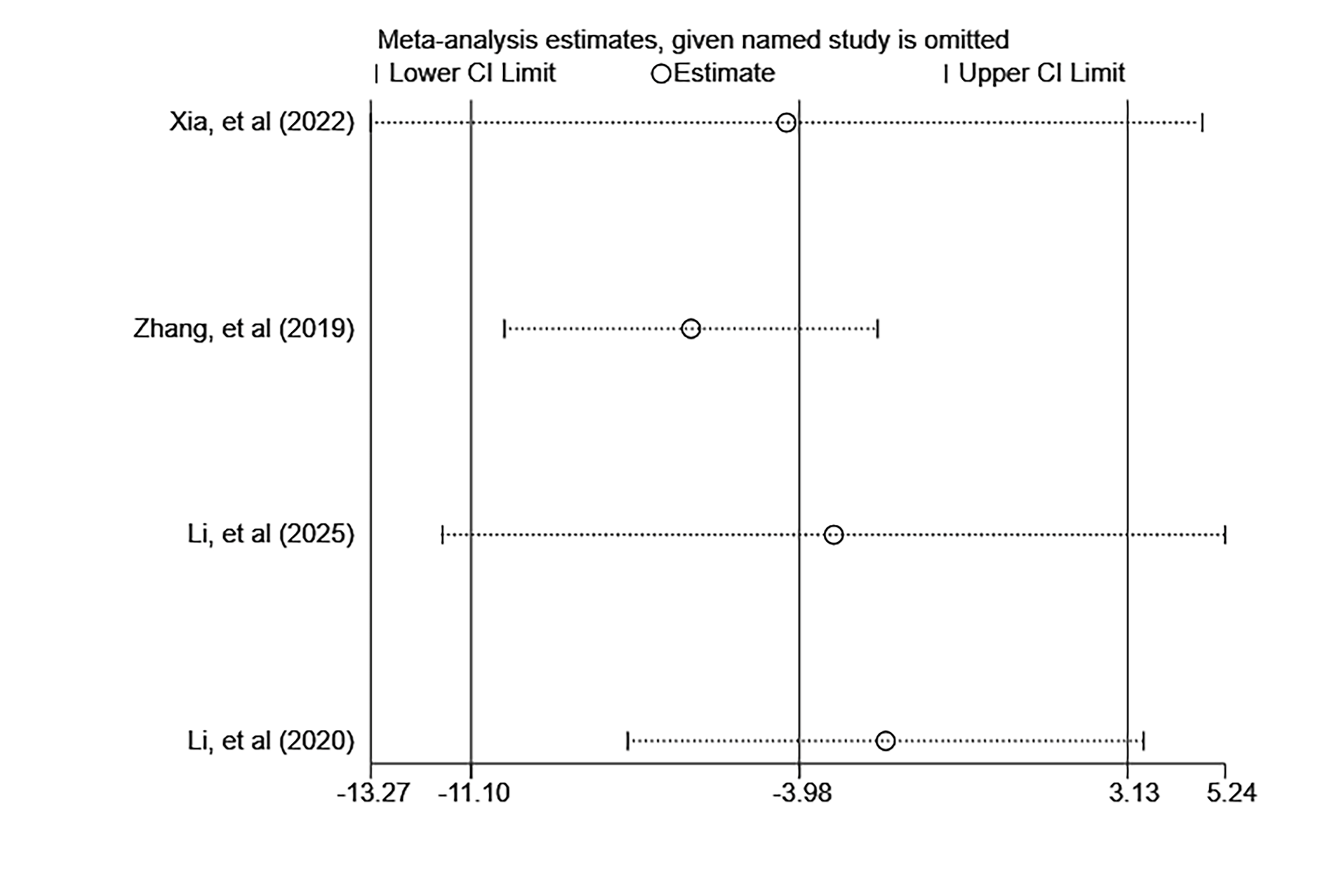


Sensitivity analyses of CD8⁺ T lymphocyte ratio


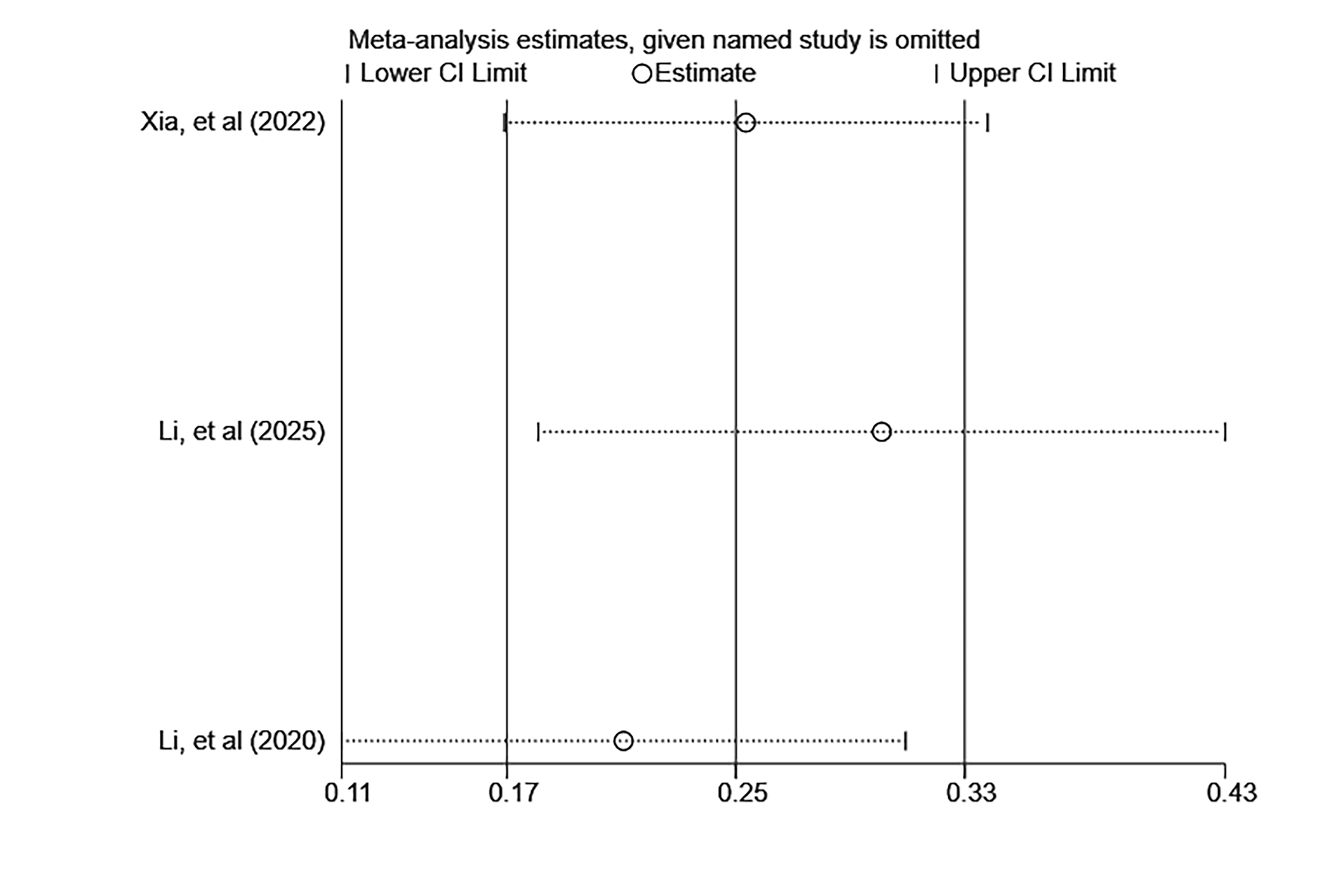


Sensitivity analyses of CD4⁺/CD8⁺ ratio


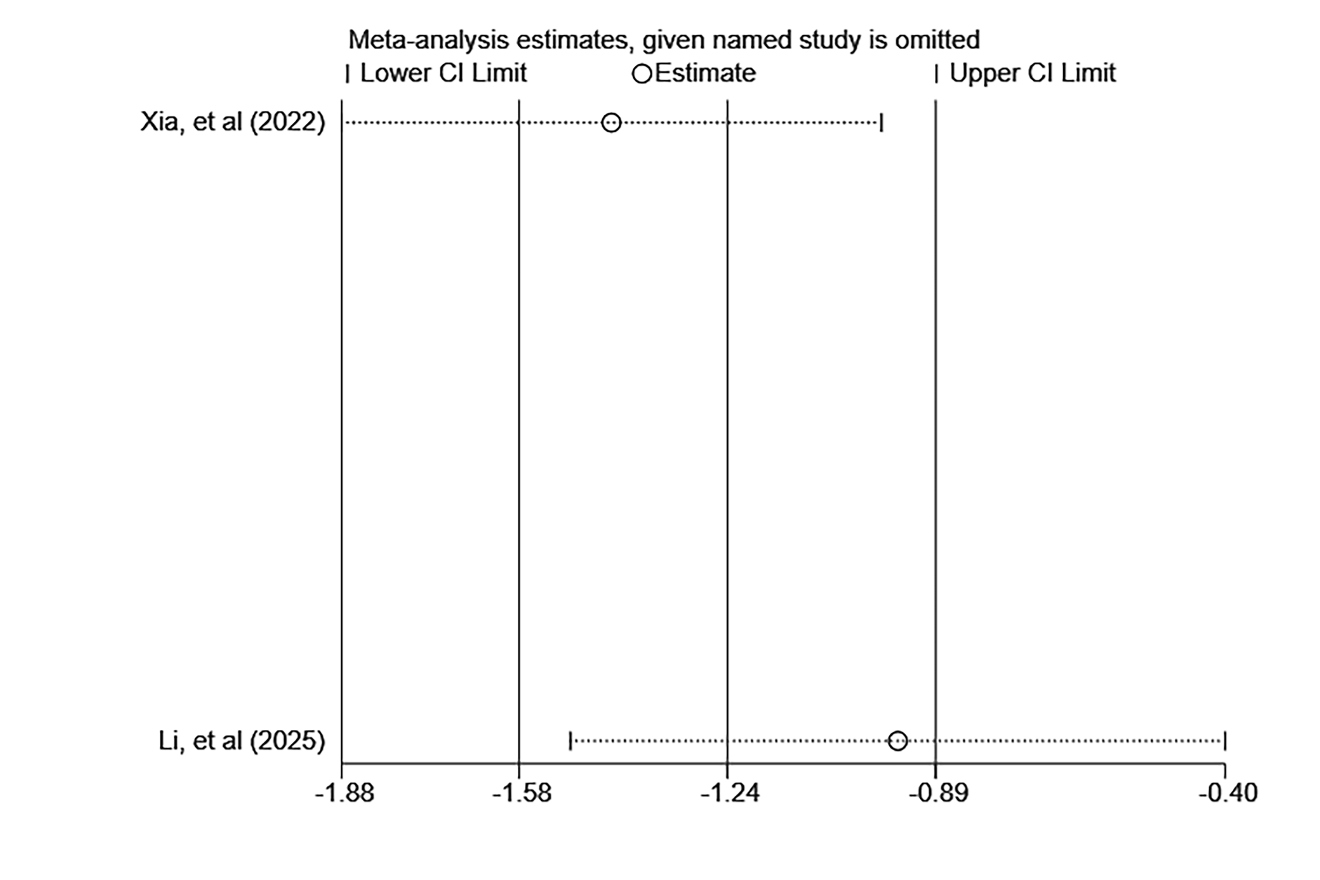


Sensitivity analyses of CEA


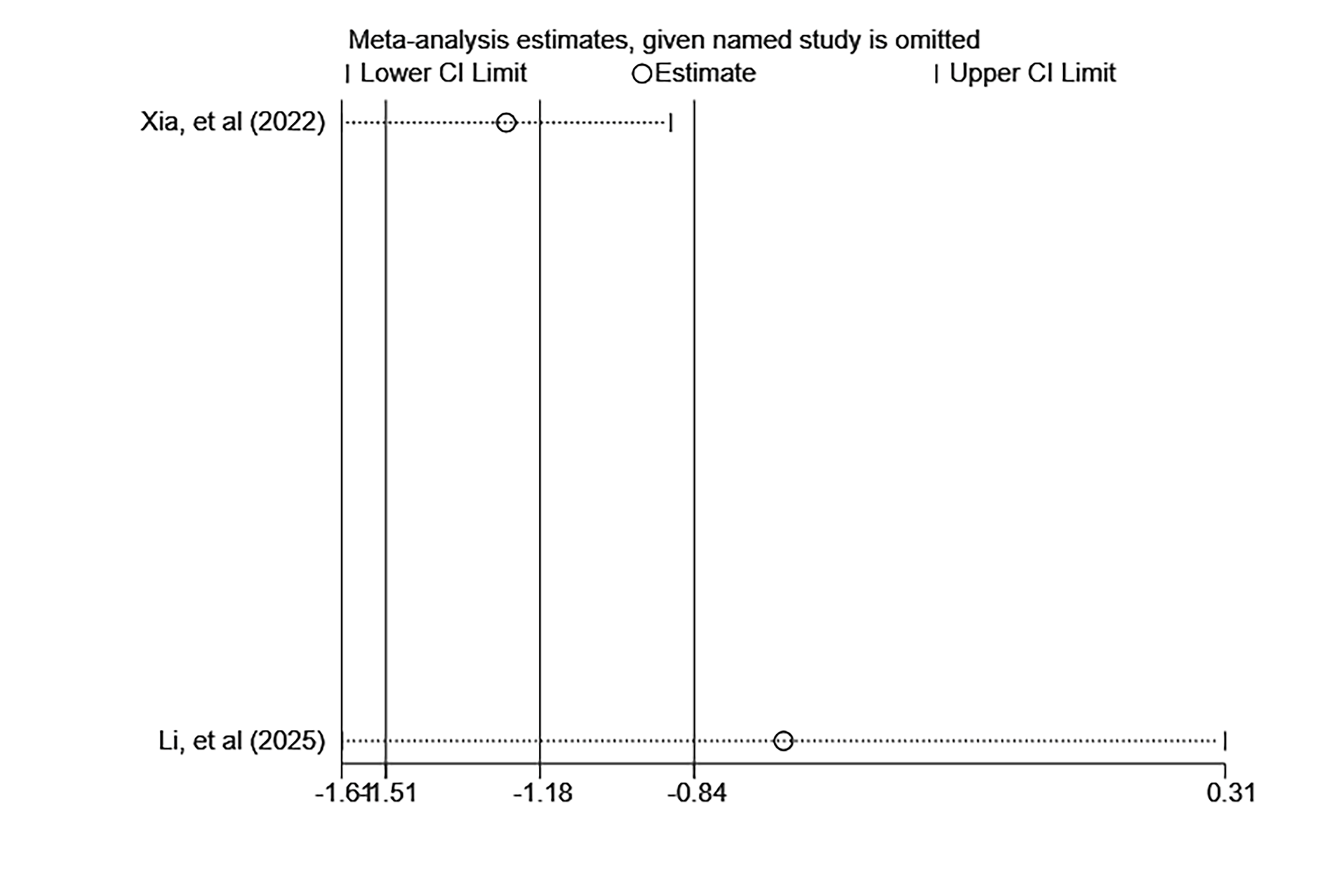


Sensitivity analyses of SCC


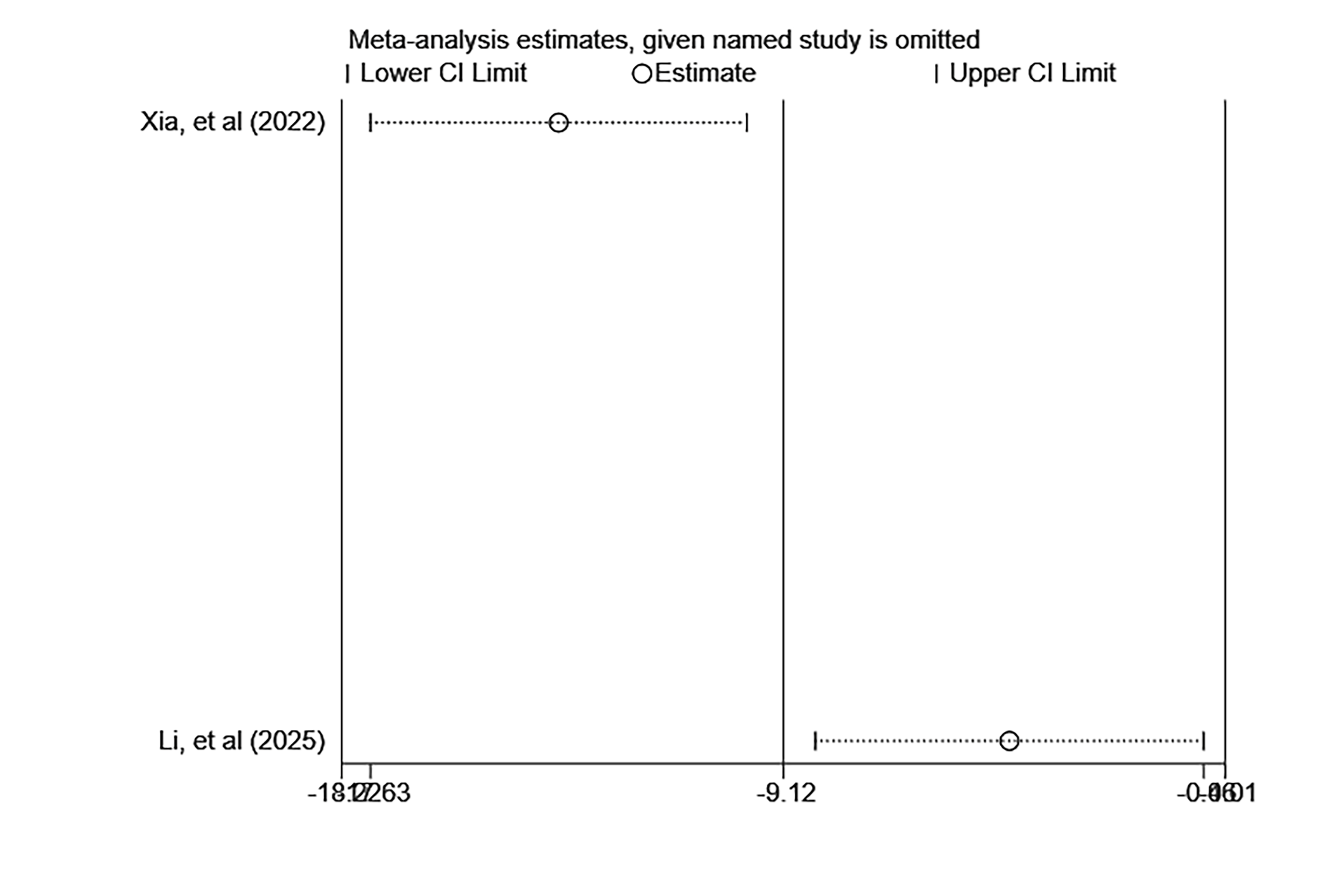


Sensitivity analyses of CA125


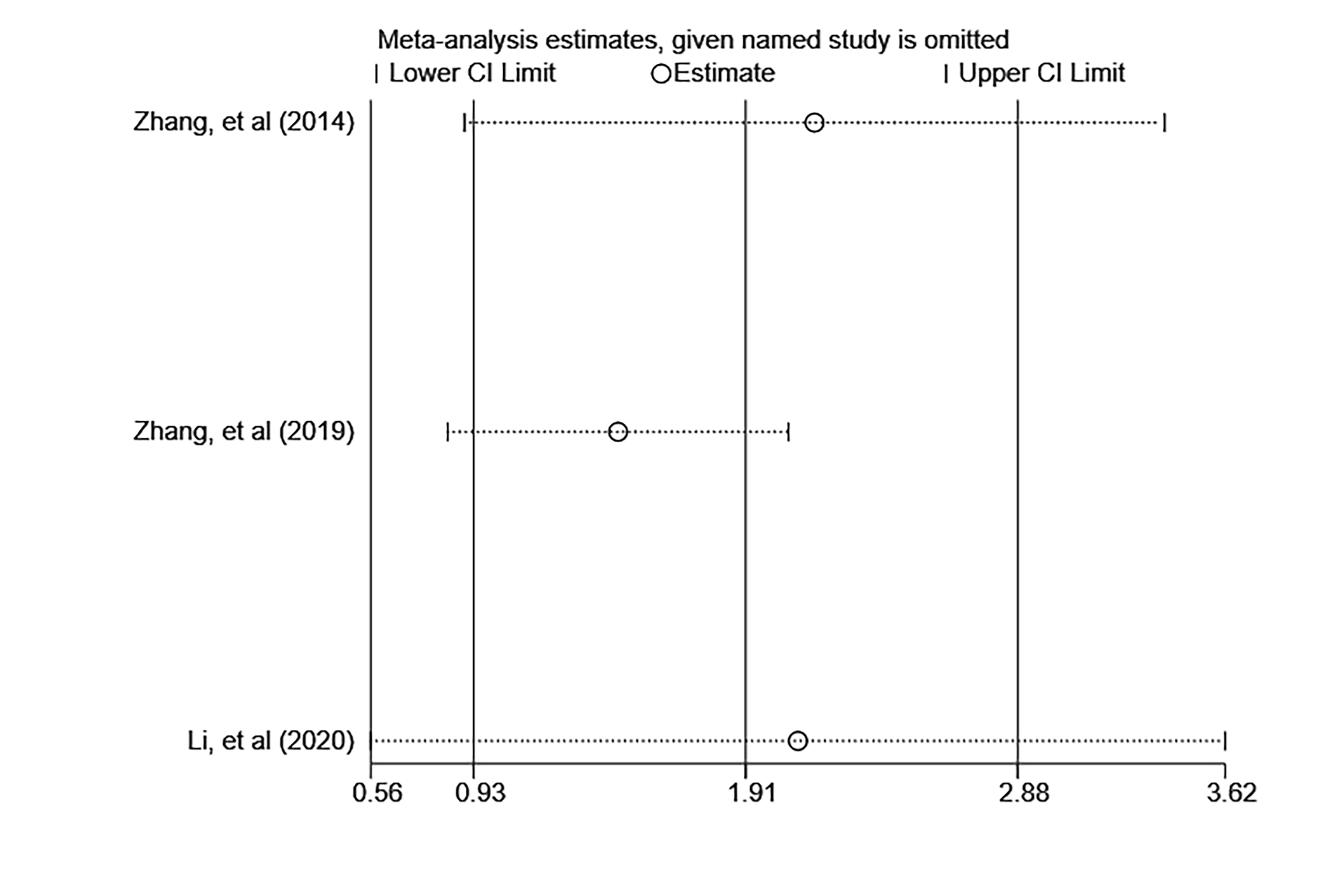


Sensitivity analyses of white blood cells


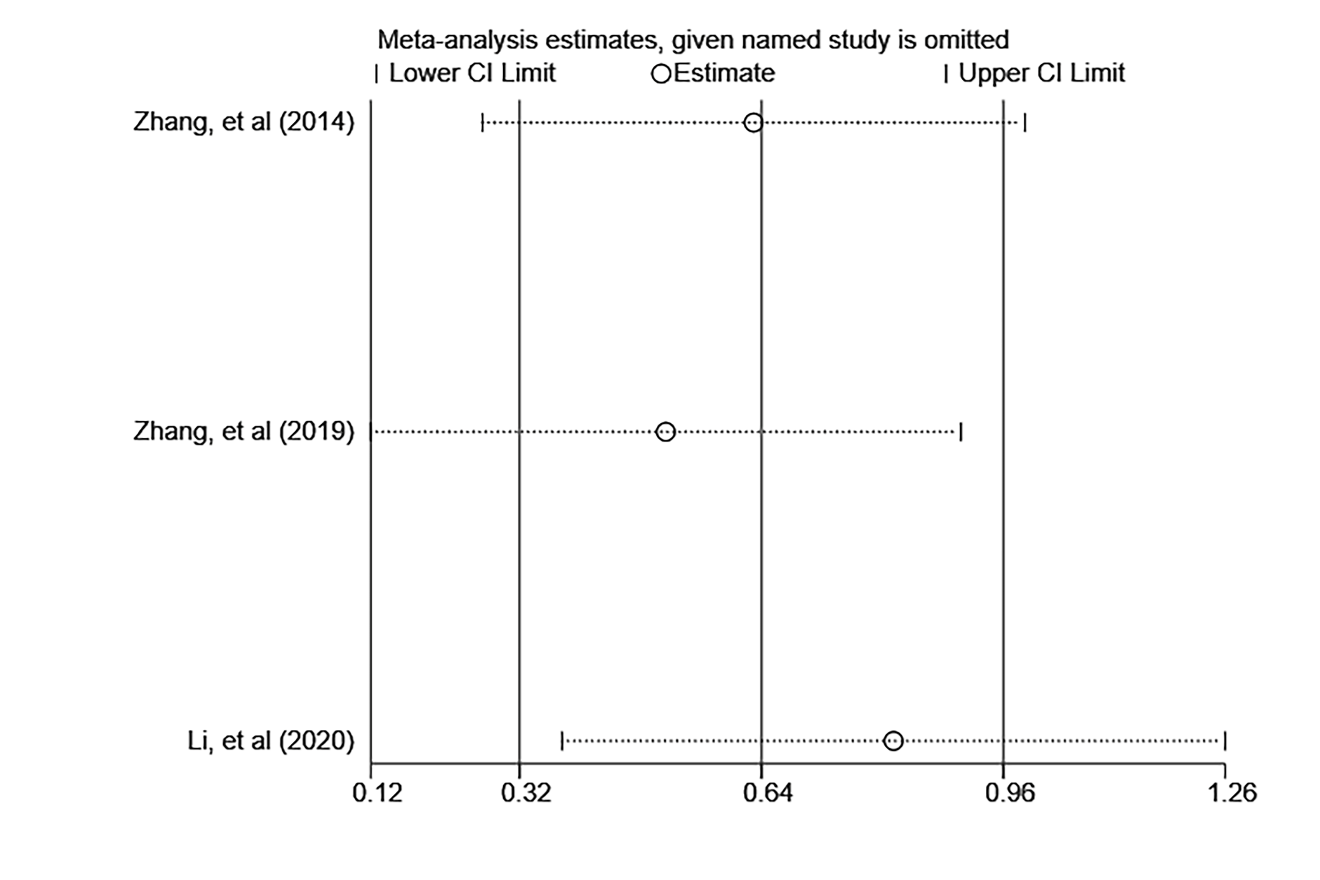


Sensitivity analyses of red blood cells


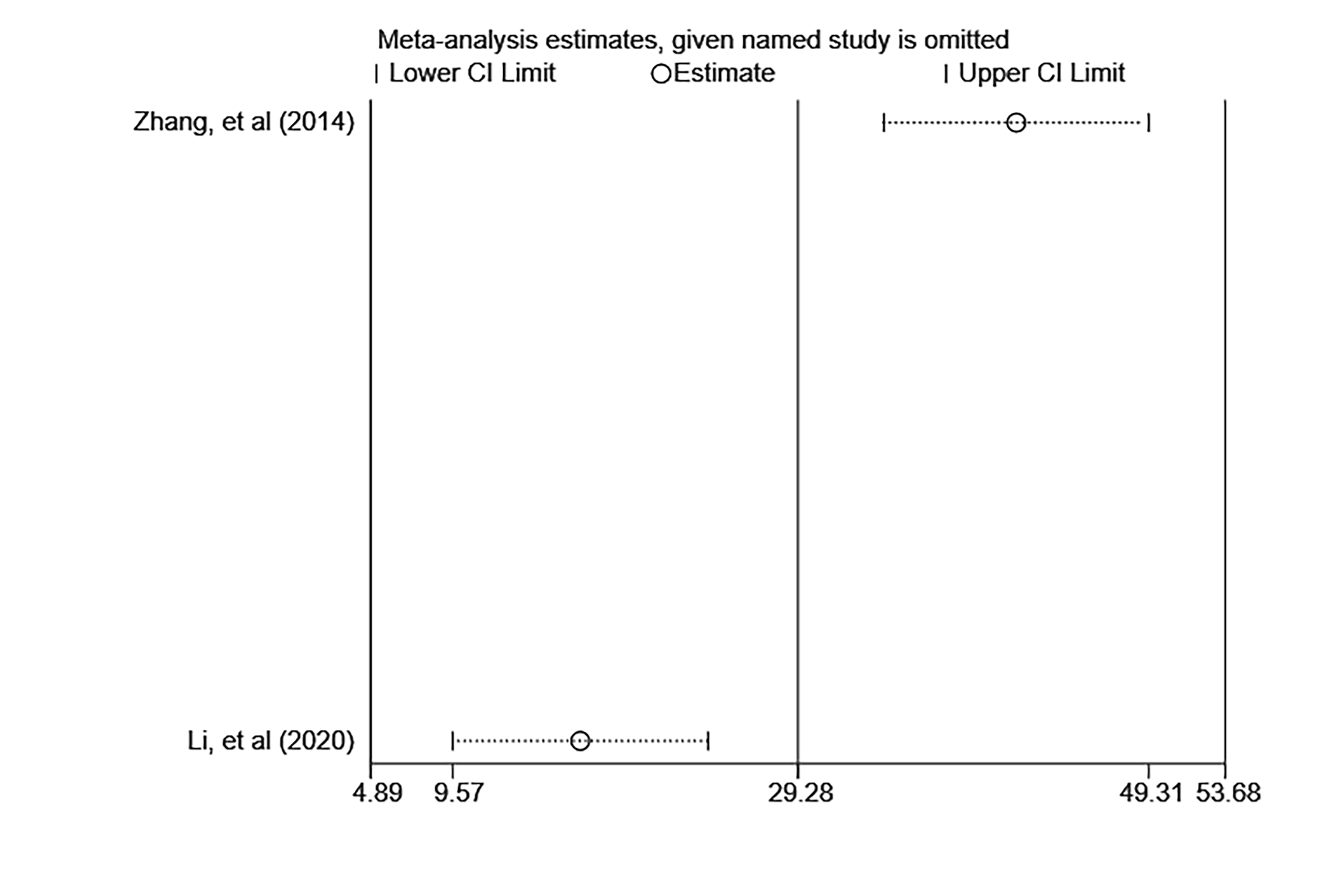


Sensitivity analyses of platelets


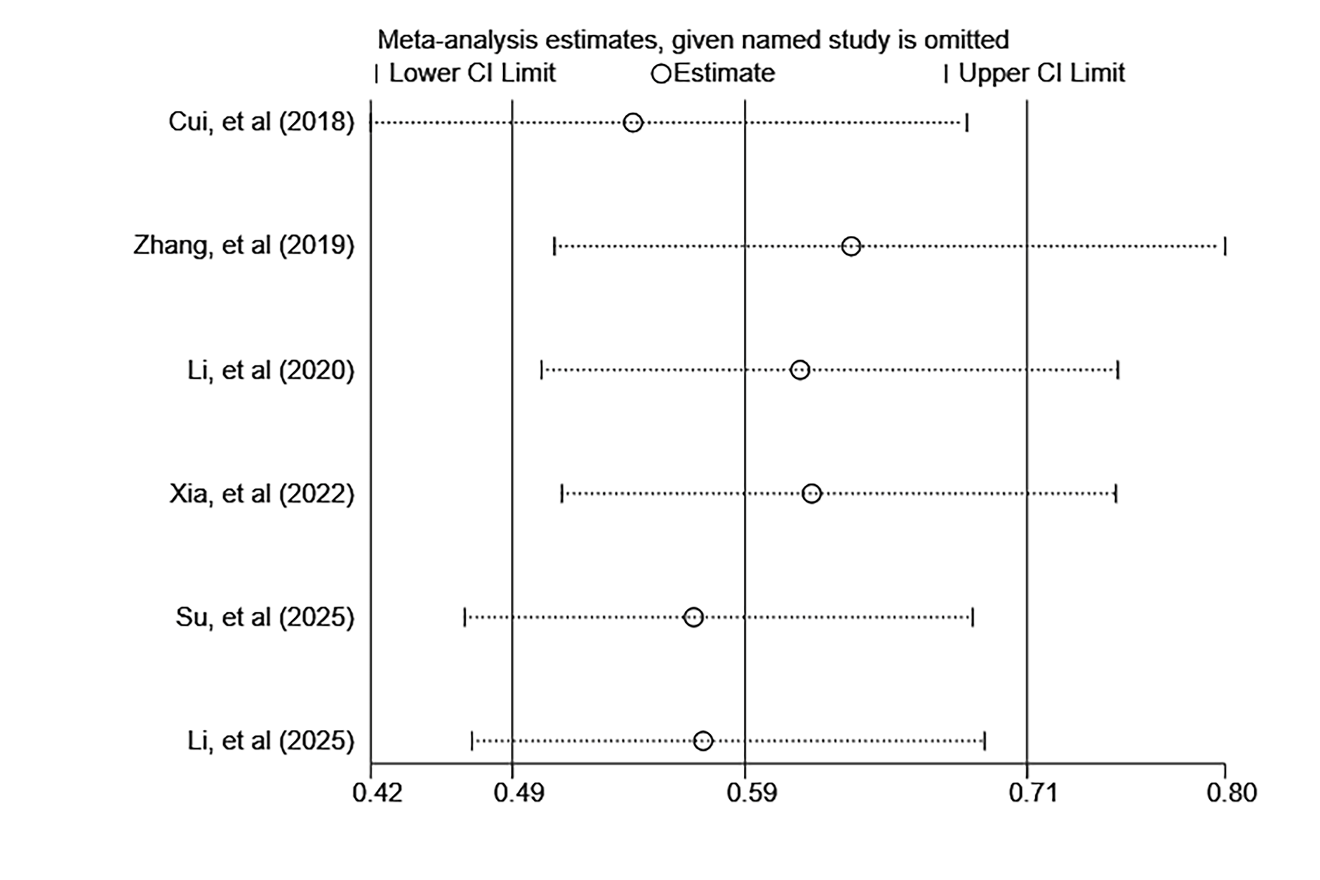


Sensitivity analyses of gastrointestinal reactions


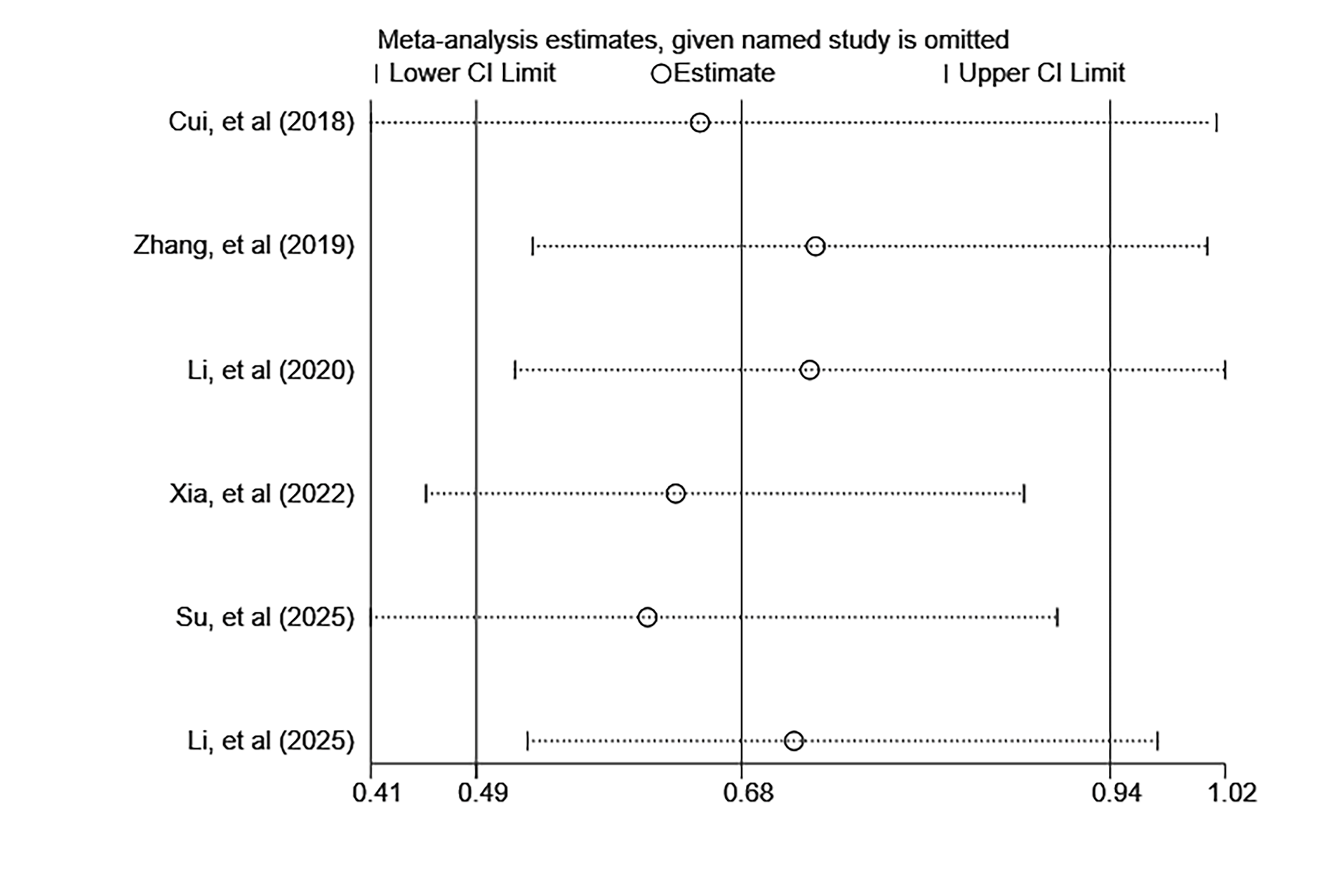


Sensitivity analyses of myelosuppression


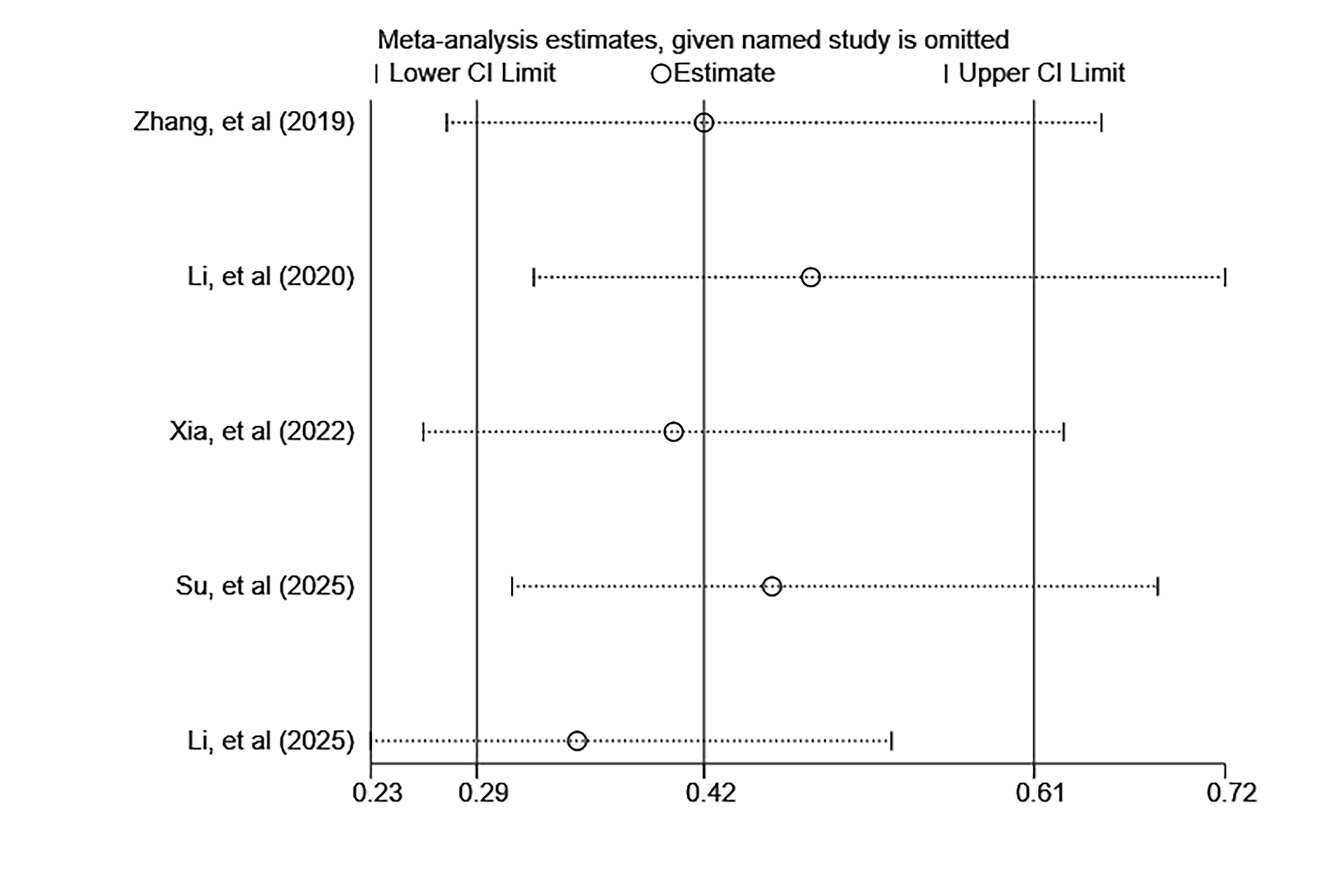


Sensitivity analyses of liver and kidney damage
