# Supplementary material for: Astragalus polysaccharides combined with radiochemotherapy for cervical cancer: a systematic review and meta-analysis of randomized controlled studies
Source: Front Pharmacol. 2025 Nov 11;16:1699902. doi: 10.3389/fphar.2025.1699902 (PMC12643993; doi:10.3389/fphar.2025.1699902)
Supplement: Supplementary file 3 [file Supplementaryfile2.docx]

**Table 2:** Summary of evidence grades based on the GRADE System

**Table 2 (a)**

| Outcome and follow-up | Patients (studies), N | Relative effect (95% CI) | **Absolute effects (95% CI)** | | | Certainty |
| --- | --- | --- | --- | --- | --- | --- |
|  |  |  | **Chemotherapy and radiation therapy** | **APS + Chemotherapy and radiation therapy** | **Difference** |  |
| ORR | 676 (8 RCTs) | **RR = 1.43** (1.24 to 1.64) | 456 per 1,000 | **652 per 1,000** (565 to 747) | **196 more per 1,000** (from 109 more to 292 more) | ⨁⨁◯◯ Low^a, b^ |
| DCR | 676 (8 RCTs) | **RR = 1.16** (1.08 to 1.24) | 769 per 1,000 | **892 per 1,000** (831 to 954) | **123 more per 1,000** (from 62 more to 185 more) | ⨁⨁◯◯ Low^a, b^ |
| CD3⁺ T lymphocyte ratio | 330 (3 RCTs) | - | 0 | - | **14.51** (1.64 to 27.39) | ⨁◯◯◯ Very low^c, d, e, f^ |
| CD4⁺ T lymphocyte ratio | 412 (4 RCTs) | - | 0 | - | **4.87** (1.79 to 7.96) | ⨁⨁◯◯ Low^c, d^ |
| CD8⁺ T lymphocyte ratio | 412 (4 RCTs) | - | 0 | - | **-3.98** (-11.1 to 3.13) | ⨁◯◯◯ Very low^c, d, e^ |
| CD4⁺/CD8⁺ ratio | 312 (3 RCTs) | - | 0 | - | **0.25** (0.17 to 0.33) | ⨁⨁◯◯ Low^f, g^ |
| CEA | 182 (2 RCTs) | - | 0 | - | **-1.24** (-1.58 to -0.89) | ⨁⨁◯◯ Low^f, g^ |
| SCC | 182 (2 RCTs) | - | 0 | - | **-1.18** (-1.51 to -0.84) | ⨁⨁◯◯ Low^f, g^ |
| CA125 | 182 (2 RCTs) | - | 0 | - | **-9.12** (-18.22 to -0.01) | ⨁◯◯◯ Very low^d, e, f, g^ |
| **CI:** confidence interval; **MD:** mean difference; **RR:** risk ratio | | | | | | |

**Explanations:**

a. 3 studies did not explicitly describe the randomization method, and none of the studies mentioned the implementation of allocation concealment or blinding.

b. small sample sizes.

c. 1 studies did not explicitly describe the randomization method, and none of the studies mentioned the implementation of allocation concealment or blinding.

d. High heterogeneity, I² > 75%.

e. Excessively wide confidence intervals.

f. Limited number of studies.

g. None of the studies mentioned the implementation of allocation concealment or blinding.

**Table 2 (b)**

| Outcome and follow-up | Patients (studies), N | Relative effect (95% CI) | **Absolute effects (95% CI)** | | | Certainty |
| --- | --- | --- | --- | --- | --- | --- |
|  |  |  | **Chemotherapy and radiation therapy** | **APS + Chemotherapy and radiation therapy** | **Difference** |  |
| white blood cells | 270 (3 RCTs) | - | 0 | - | **1.91** (0.93 to 2.88) | ⨁◯◯◯ Very low^a, b, c^ |
| red blood cells | 270 (3 RCTs) | - | 0 | - | **0.64** (0.32 to 0.96) | ⨁⨁◯◯ Low^a, c^ |
| platelets | 170 (2 RCTs) | - | 0 | - | **29.28** (4.89 to 53.68) | ⨁◯◯◯ Very low^b, c, d^ |
| KPS score | 154 (2 RCTs) | - | 0 | - | **6.64** (4.12 to 9.16) | ⨁⨁◯◯ Low^c, e^ |
| **CI:** confidence interval | | | | | | |

**Explanations:**

a. 2 studies did not explicitly describe the randomization method, and none of the studies mentioned the implementation of allocation concealment or blinding.

b. High heterogeneity, I² > 50%.

c. Limited number of studies.

d. 1 studies did not explicitly describe the randomization method, and none of the studies mentioned the implementation of allocation concealment or blinding.

e. Neither study mentioned the implementation of allocation concealment or blinding.

**Table 2 (c)**

| Outcome and follow-up | Patients (studies), N | Relative effect (95% CI) | **Absolute effects (95% CI)** | | | Certainty |
| --- | --- | --- | --- | --- | --- | --- |
|  |  |  | **Chemotherapy and radiation therapy** | **APS + Chemotherapy and radiation therapy** | **Difference** |  |
| gastrointestinal reactions | 566 (6 RCTs) | **RR = 0.59** (0.49 to 0.71) | 523 per 1,000 | **309 per 1,000** (256 to 371) | **214 fewer per 1,000** (from 267 fewer to 152 fewer) | ⨁⨁◯◯ Low^a, b^ |
| myelosuppression | 566 (6 RCTs) | **RR = 0.65** (0.51 to 0.84) | 661 per 1,000 | **430 per 1,000** (337 to 555) | **231 fewer per 1,000** (from 324 fewer to 106 fewer) | ⨁◯◯◯ Very low^a, c^ |
| hepatic or renal dysfunction | 498 (5 RCTs) | **RR = 0.42** (0.29 to 0.61) | 305 per 1,000 | **128 per 1,000** (89 to 186) | **177 fewer per 1,000** (from 217 fewer to 119 fewer) | ⨁⨁◯◯ Low^a, b^ |
| **CI:** confidence interval; **RR:** risk ratio | | | | | | |

**Explanations:**

a. 1 studies did not explicitly describe the randomization method, and none of the studies mentioned the implementation of allocation concealment or blinding.

b. Moderate heterogeneity among studies, 0% < I² < 50%.

c. High heterogeneity, 50% < I² < 75%.
